# Estrogenic in vitro evaluation of zearalenone and its phase I and II metabolites in combination with soy isoflavones

**DOI:** 10.1007/s00204-022-03358-3

**Published:** 2022-08-20

**Authors:** Dino Grgic, Andrea Betschler, Rebeka Früholz, Barbara Novak, Elisabeth Varga, Doris Marko

**Affiliations:** 1 Department of Food Chemistry and Toxicology, Faculty of Chemistry, University of Vienna, Währinger Str. 38-40, 1090 Vienna, Austria; 2 Doctoral School in Chemistry, University of Vienna, Währinger Str. 38-40, 1090 Vienna, Austria; 3 DSM, BIOMIN Research Center, Technopark 1, 3430 Tulln, Austria

**Keywords:** Isoflavones, Zearalenone, Phytoestrogens, Mycoestrogens, Combinatory toxicology, Endocrine disruptors

## Abstract

Humans and animals are exposed to multiple substances in their food and feed that might have a negative health impact. Among these substances, the *Fusarium* mycoestrogen zearalenone (ZEN) and its metabolites α-zearalenol (α-ZEL) and α-zearalanol (α-ZAL) are known to possess endocrine disruptive properties. In a mixed diet or especially animal feed, these potential contaminants might be ingested together with naturally occurring phytoestrogens such as soy isoflavones. So far, risk assessment of potential endocrine disruptors is usually based on adverse effects of single compounds whereas studies investigating combinatorial effects are scarce. In the present study, we investigated the estrogenic potential of mycoestrogens and the isoflavones genistein (GEN), daidzein (DAI) and glycitein (GLY) as well as equol (EQ), the gut microbial metabolite of DAI, in vitro alone or in combination, using the alkaline phosphatase (ALP) assay in Ishikawa cells. In the case of mycoestrogens, the tested concentration range included 0.001 to 10 nM with multiplication steps of 10 in between, while for the isoflavones 1000 times higher concentrations were investigated. For the individual substances the following order of estrogenicity was obtained: α-ZEL > α-ZAL > ZEN > GEN > EQ > DAI > GLY. Most combinations of isoflavones with mycoestrogens enhanced the estrogenic response in the investigated concentrations. Especially lower concentrations of ZEN, α-ZEL and α-ZAL (0.001—0.01 nM) in combination with low concentrations of GEN, DAI and EQ (0.001—0.1 µM) strongly increased the estrogenic response compared to the single substances.

**Supplementary Information:**

The online version contains supplementary material available at 10.1007/s00204-022-03358-3.

## Introduction

**Fig. 1:**
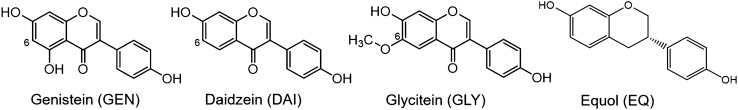
Chemical structures of glycitein (GLY), genistein (GEN), daidzein (DAI) and S-equol (EQ) (created with chem-space.com)

In the last decade, dietary habits have changed in western countries. According to an Australian survey plant-based diets gained in popularity and the number of vegetarians or vegans in the population increased (Roy Morgan 2016). For instance, the percentage of vegetarians and vegans in Germany doubled from 5 to 10% between 2020 and 2021. As a consequence, the consumption of alternatives to animal products increased (BMEL 2021). Many of these plant alternatives are based on soy products as the primary substitute for meat due to the high protein content and potential health benefits which are associated with the presence of isoflavones (ISF) like genistein (GEN), glycitein (GLY), daidzein (DAI) and its gut microbial metabolite equol (EQ) (Fig. 1) (Qin et al. 2022). Epidemiological studies in humans suggest that a high intake of ISF may be associated with several positive health effects. For example, in Asian countries where soy is part of a traditional diet, the risk of developing breast, prostate and colorectal cancers is lower compared to low soy-consuming countries (Wu et al. 2008; Yan and Spitznagel 2009; Yan et al. 2010). Other studies claim various clinical benefits of ISF intake including a reduction in the incidental onset of certain types of diseases such as coronary heart disease, osteoporosis and memory loss (Tham et al. 1998; Sarkar and Li 2003; Sacks et al. 2006; Zheng et al. 2016). However, in vitro studies indicate proliferative effects of ISF on estrogen-sensitive cancer cells (Matsumura et al. 2005). Although the health effects are still controversially discussed, ISF or ISF-containing preparations are used as hormone replacement therapy in postmenopausal women (Carusi 2000; Aguiar and Barbosa 2014).

Regarding animal feed, soy is used for decades as the main protein source due to its high protein content (Goldsmith 2008) and several beneficial properties associated with ISF, such as growth promoting, improved antioxidative and immune functions (Grgic et al. 2021). However, there are also reports which indicate that a high intake of ISF might lead to negative health effects, mainly affecting the reproductive tract of female farm animals (Bennetts et al. 1946; Hashem and Soltan 2016), e.g. infertility, uterine prolapse and swelling of mammary glands and vulva.

**Fig. 2:**
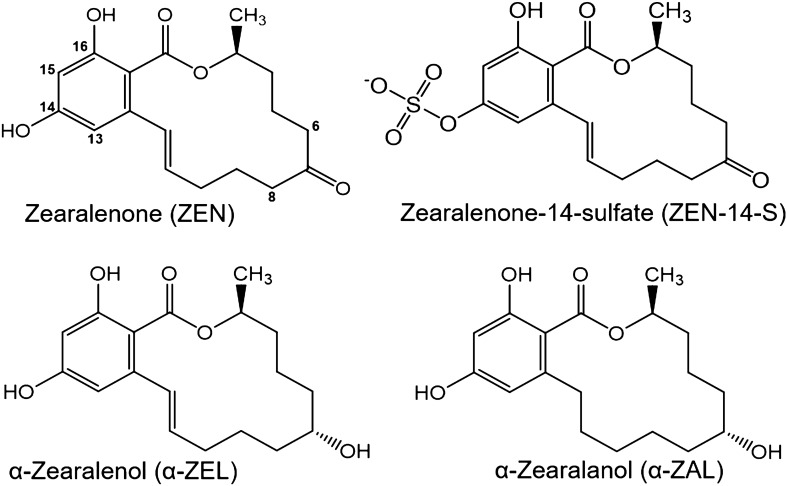
Chemical structures of zearalenone (ZEN), zearalenone-14-sulfate (ZEN-14-S), α-zearalenol (α-ZEL) and α-zearalanol (α-ZAL) (created with chem-space.com)

ISF were described to co-occur frequently together with the mycotoxin zearalenone (ZEN) and several of its metabolites (Fig. 2) (up to 60%) in various animal feeds (Grgic et al. 2021; Penagos-Tabares et al. 2021). ZEN is a secondary fungal metabolite produced by *Fusarium* species and is commonly found in grains and legumes (Grgic et al. 2021). ZEN is known to have multiple toxic effects on humans and animals (Kuiper-Goodman et al. 1987; Fink-Gremmels and Malekinejad 2007; Kowalska et al. 2016; Rogowska et al. 2019). However, its most prominent effect is its ability to act as an endocrine disruptor. Its reduced phase I metabolite α-zearalenol (α-ZEL) is known to have even stronger estrogenic effects, whereas its phase II metabolites like the plant metabolite ZEN-14-glucoside are described to have lost their estrogenic properties (Molina-Molina et al. 2014; Dellafiora et al. 2017).

**Fig. 3:**
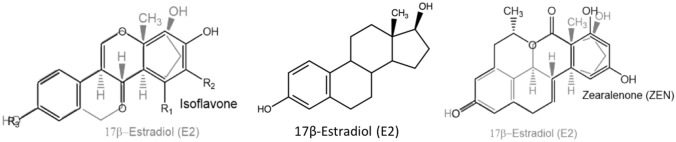
Chemical structure of zearalenone (ZEN) and isoflavone (ISF) scaffold in comparison, with 17-β-estradiol (E2) (created with chem-space.com)

Both, ISF and ZEN represent xenoestrogens based on the structural and functional similarity to the endogenous hormone 17-β-estradiol (E2) (Fig. 3), which eventually bind and activate the estrogen receptors (ER), but with different affinity to the different ER isoforms α and β. ZEN is known to favorably bind to the ERα, whereas ISF have a higher affinity to the ERβ (Kuiper et al. 1998). The different binding affinity properties to the ERs of these two classes of substances might cause an enhanced estrogenic effect when they occur together (Vejdovszky et al. 2017b).

According to the scientific opinion of the European Food Safety Authority (EFSA) from 2011, ZEN exposure is quite high for certain populations and these values might be even higher for vegetarians and vegans (EFSA 2011). Therefore, regulatory limits for ZEN in different food and feed stuff have been established. For instance, in maize intended for direct human consumption and for feed designed for piglets this value should not exceed 100 µg/kg (EFSA 2011; European Commission 2016, 2022). However, risk assessments are still predominantly based on the toxicological data of single substances, which often underestimate the toxicological potential of certain mixtures. Data on combinatorial effects are still scarce and therefore, to ensure extensive health protection and food safety, further studies are required. Our aim was to provide a more detailed insight into the estrogenic properties of combinations between the mycoestrogen ZEN and its metabolites, with the phytoestrogens GEN, GLY, DAI and EQ.

## Material and methods

### Materials

Cell culture 96-well plates and flasks were obtained from Sarstedt (Nürnbrecht, Germany). Cell culture media (Minimal Essential Medium (MEM) and Dulbecco’s Modified Eagle Medium/F12 (DMEM/F-12) without phenol red) and supplements (fetal bovine serum (FBS), charcoal-stripped FBS (CD-FBS), L-glutamine and penicillin–streptomycin (P/S)) were purchased from Gibco, Thermo Fisher Scientific, (Waltham/MA, USA). Zearalenone (ZEN), α-zearalenol (α-ZEL), α-zearalanol (α-ZAL), 17-β-estradiol (E2), 4-nitrophenylphosphate, diethanolamine, magnesium chloride and sulforhodamine B (SRB) were obtained from Sigma Aldrich Chemie GmbH (Schnelldorf, Germany), whereas daidzein (DAI), equol (EQ), genistein (GEN) and glycitein (GLY) were purchased from Extrasynthese (Genay Cedex, France). Dimethly sulfoxide (DMSO), NaCl, KCl, Na_2_HPO_4_, Na_2_HPO_4_ × 2 H_2_O and KH_2_PO_4_ were purchased from Roth (Karlsruhe, Germany). ZEN-4-sulfate ammonium salt was obtained from Santa Cruz Biotechnology (Dallas/TX, USA) and is the same compound as ZEN-14-sulfate (ZEN-14-S) using the newer International Union of Pure and Applied Chemistry (IUPAC) numbering system (Metzler 2011). The CellTiter-Blue® Cell Viability Assay Kit was purchased from Promega Corporation (Madison/WI, USA).

### Cell line

The human endometrial adenocarcinoma cell line “Ishikawa” was purchased from the European Collection of Authenticated Cell Cultures (ECACC, Wiltshire, United Kingdom). Cell stocks were stored in liquid nitrogen containers and two weeks prior to the start of the in vitro experiments, cells were taken in culture. They were cultivated in an incubator at 37 °C with 5% CO_2_ and 95% humidity using the growth medium consisting of MEM, supplemented with 5% (*v/v*) heat-inactivated FBS, 1% L-glutamine and 1% P/S. Cells of passage number 5 were split at a confluency of about 80% and kept in culture up to the maximum passage number of 40. Before the assays were performed, the growth medium was removed and replaced with the assay medium consisting of DMEM/F-12 supplemented with 5% CD-FBS and 1% P/S.

### Alkaline phosphatase assay

The assays were performed in 96-well plates and per well, 15 000 or 10 000 Ishikawa cells in the assay medium were seeded and grown for 24 or 48 h, respectively. Incubation was carried out for 48 h with different concentrations of ZEN, α-ZEL, α-ZAL, ZEN-14-S, GEN, DAI, EQ and GLY as single substances or in the respective combinations at different concentrations. The substances were solved in DMSO in 200 times higher concentrations than the tested concentration, followed by dilution in the assay medium. In the case of single substance testing and for solvent control, DMSO was added to reach 1% in the final incubation solutions. The concentration ranges were 0.001 to 10 nM in the case of ZEN-metabolites and 0.001 to 10 µM in the case of ISF, with 1:10 dilution steps in between. An exception was GLY where the following concentrations were applied 0.01, 0.1, 1, 10 and 20 µM. E2 (1 nM) served as a positive control. All experiments were performed at least in five independent biological replicates (measurements with different cell passages) with technical triplicates (repeated measurements with the same cell passage) each. Since each ISF was measured with every mycoestrogen and the single substances were included on all plates of the respective combinations, at least 20 biological replicates of the respective single substance in technical triplicates were obtained.

Following the 48 h incubation, the supernatants were discarded and all wells were washed three times with 150 µL phosphate-buffered saline (PBS) per well. After the PBS was completely removed, the plate was placed in the freezer at −80 °C for at least 20 min. During this procedure, the cells were lysed, and the alkaline phosphatase (ALP) was released. The plate was thawed for five minutes at room temperature before 50 µL of the ALP buffer (containing 5 mM 4-nitrophenylphosphate, 1 M diethanolamine and 0.24 mM MgCl_2_) was added in the dark. After five minutes at room temperature, the plate was placed in the plate reader and the absorbance was measured at 405 nm every 2 min for 1 h at 37 °C using a Victor V3 1240 Multilabel Counter from Perkin Elmer (Waltham/MA, USA) or a Cytation 3 Cell Imaging Multi-Mode Reader from Biotek® (Winooski/VM, USA). The activity of the ALP was calculated as the slope of the curve, obtained by the measurements monitored over 1 h. Final results were referred to the solvent and positive control which were set to 0 and 100%, respectively.

### Coupled CellTiter-Blue® and SRB cytotoxicity assay

After cell seeding (15 000 or 10 000 cells/well) and growing for 24 h or 48 h, respectively, the cells were incubated with the incubation solutions. These were prepared as described for the ALP assay and the same single substances and combinations thereof were incubated for 48 h. As solvent control 1% DMSO was used. All experiments were performed at least in five independent biological replicates with technical triplicates each, resulting in at least 20 biological replicates for the single substances. After the incubation time (48 h) the following steps were carried out in the dark. The medium was aspirated and 100 µL of a CellTiter-Blue® (CTB) incubation solution (1:10 dilution of CTB reagent and DMEM/F-12 (5% CD-FBS, 1% P/S)) was added. After an additional incubation time of 50 min with the CTB solution, 90 µL of each well were transferred to a new, black 96-well plate. The black plate was directly measured by an excitation wavelength of 560 nm and emission of 590 nm with a gain of 65 using the Victor V3 1240 Multilabel Counter from Perkin Elmer (Waltham/MA, USA) or the Cytation 3 Cell Imaging Multi-Mode Reader from Biotek® (Winooski/VT, USA). Final results were referred to as the solvent control in percentage.

The remaining 10 µL of the initial 96-well plate were aspirated, and cells were fixed with 10 µL of a 50% (*w/v*) trichloroacetic acid solution in distilled water for one hour at 4 °C. Thereafter, the 96-well plate was washed four times with tap water and dried overnight in the dark. Afterwards, 50 µL of the SRB solution (4 g SRB reagent solved in 1 L distilled water containing 1% acetic acid) were added to each well. After one-hour staining in the dark at room temperature, the coloring solution was discarded, and the plate was washed twice with tap water and twice with 1% acetic acid. After another drying step overnight in the dark, the dye was dissolved under alkaline conditions in 100 µL Tris base (0.30 g tris(hydroxymethyl-) aminomethane solved in 250 mL distilled water) by shaking for 5 min in the plate reader (Victor V3 1240 Multilabel Counter from Perkin Elmer (Waltham/MA, USA) or the Cytation 3 Cell Imaging Multi-Mode Reader from Biotek® (Winooski/VT Vermont, USA)). Subsequently, the absorbance was measured at 570 nm. As for the CTB, the final results were referred to the solvent control in percentage.

### Statistics

Measurements of single substances and their combinations for the ALP and the cytotoxicity assays were conducted each in technical triplicates and in at least five independent biological replicates (*n* ≥ 5).

Biological replicates were tested for outliers according to Nalimov and for normality with the Kolmogorov–Smirnov test. Identified outliers were excluded in the calculation of the mean values and the standard deviations. At maximum, three outliers were excluded for the single substances resulting in at least 17 biological replicates and a maximum of one outlier was excluded for the combinations resulting in at least 4 biological replicates.

Statistical analyses and plotting of data were performed with the software Origin Pro® 2021, with significance levels of 5%, 1% and 0.1%, respectively (#, *x* = *p* < 0.05; ##, *xx* = *p* < 0.01; ###, *xxx* = *p* < 0.001). Significant differences were evaluated via one-way analysis of variance (ANOVA) followed by Fisher’s least significant difference (LSD) post hoc test. Cytotoxicity results were evaluated by using one-way and two-way Student’s *t*-test.

### Combination Index (CI)

To assess possible interactions between multiple compounds, like synergistic, antagonistic or additive effects, the mathematical combination index (CI) method developed by Chou and Talalay (Chou and Talalay 1981) was used. The CI is based on a variety of statistical equations including the mass-action law, the Michaelis–Menten equation (for enzyme kinetics), the Henderson-Hasselbalch equation (for pH ionization), the Hill equation (for ligand binding saturation) and the Scatchard equation (for receptor binding). The basis of the calculation of the CI is the median effect equation (MEE) (see Formula 1), which describes the relationship between dose and effect (Chou 2006).

1 $$\frac{{{f}}_{\text{a}}}{{{f}}_{\text{u}}}\text{=}({\text{}\frac{{D}}{{{D}}_{\text{m}}}\text{)}}^{\text{m}}$$

*f*_*a*_: affected fraction by the dose.

*f*_*u*_: unaffected fraction = 1–*f*_*a*_.

*D*: dose.

*D*_*m*_: dose at mean effect (e.g., half-maximal effect concentration (EC_50_)).

*m*: coefficient standing for the shape of the relationship between dose and effect, whereby *m* = 1 indicates hyperbolic, *m* > 1 sigmoidal and *m* < 1 flat sigmoidal dose-effect curves.

To calculate the CI, the MEE is transformed into the following equation (Chou 2006):

2 $$\text{CI = }\frac{{{(D)}}_{1}}{{\text{(}{{D}}_{\text{x}}\text{)}}_{1}}+ \text{ } \frac{{{(D)}}_{2}}{{\text{(}{{D}}_{\text{x}}\text{)}}_{2}}= \text{ } \frac{{{(D)}}_{1}}{{{\text{(}{{D}}_{\text{m}}\text{)}}_{1}\text{[}\frac{{{f}}_{\text{a}}}{\text{1- }{{f}}_{\text{a}}}\text{]}}^{\frac{1}{{\text{m}}}}}+ \text{ } \frac{{{(D)}}_{2}}{{{\text{(}{{D}}_{\text{m}}\text{)}}_{2}\text{[}\frac{{{f}}_{\text{a}}}{\text{1- }{{f}}_{\text{a}}}\text{]}}^{\frac{1}{{\text{m}}}}}$$

*f*_*a*_: affected fraction by the dose.

*D*: dose.

(*D*_*x*_)_1_: dose of single substance 1 that has the same effect as the combination of (*D*)_1_+(*D*)_2_.

(*D*_*x*_)_2_: dose of single substance 2 that has the same effect as the combination of (*D*)_1_+(*D*)_2_

*D*_*m*_: dose at mean effect (e.g., ED_50_).

*m*: coefficient standing for the shape of the relationship between dose and effect, whereby *m* = 1 indicates hyperbolic, *m* > 1 sigmoidal and *m* < 1 flat sigmoidal dose-effect curves.

By calculating the CI, a statement about the effect induced by several compounds can be made. A CI value of 1 indicates an additive effect, whereas CI < 1 indicates synergism and CI > 1 antagonism (Chou 2006). The more detailed subdivision of synergism and antagonism according to Chou is illustrated in Table 1 (Chou 2006).

**Table 1:** Description of synergism and antagonism in combination studies with the combination index (CI)

Range of CI	Description
< 0.10	Very strong synergism
0.10–0.30	Strong synergism
0.30–0.70	Synergism
0.70–0.85	Moderate synergism
0.85–0.90	Slight synergism
0.90–1.10	Nearly additive
1.10–1.20	Slight antagonism
1.20–1.45	Moderate antagonism
1.45–3.30	Antagonism
3.30–10.0	Strong antagonism
> 10.0	Very strong antagonism

For applying the CI some requirements have to be fulfilled. Besides a monotonic dose–response curve, constant ratios of combinations should be used to receive the most exact evaluation of the CI. Furthermore, it is important that the maximum effects are the same for all combinations (Chou 2006; Vejdovszky et al. 2017b). Therefore, the highest ALP concentration of 115%, achieved by the combination of 0.01 nM ZEN with 1 µM EQ was set to 1. Since the CI analysis is only feasible with relative values between 0 and 1, all effects were relativized to the lowest and highest value of −26% and 115%, respectively.

## Results

### Alkaline Phosphatase Assay (ALP)

Ishikawa cells were incubated for 48 h with different concentrations ranging from 0.001 to 10 nM in case of ZEN and its metabolites and 0.001 to 10 µM in case of ISF, with 1:10 dilution steps in between. An exception was GLY due to its low estrogenicity and instead of 0.001 µM, 20 µM was included in the assessments. As positive control 1 nM E2 was used and 1% DMSO served as solvent control and their effects on ALP induction were set to 100% and 0%, respectively.

### ALP single substances

**Fig. 4:**
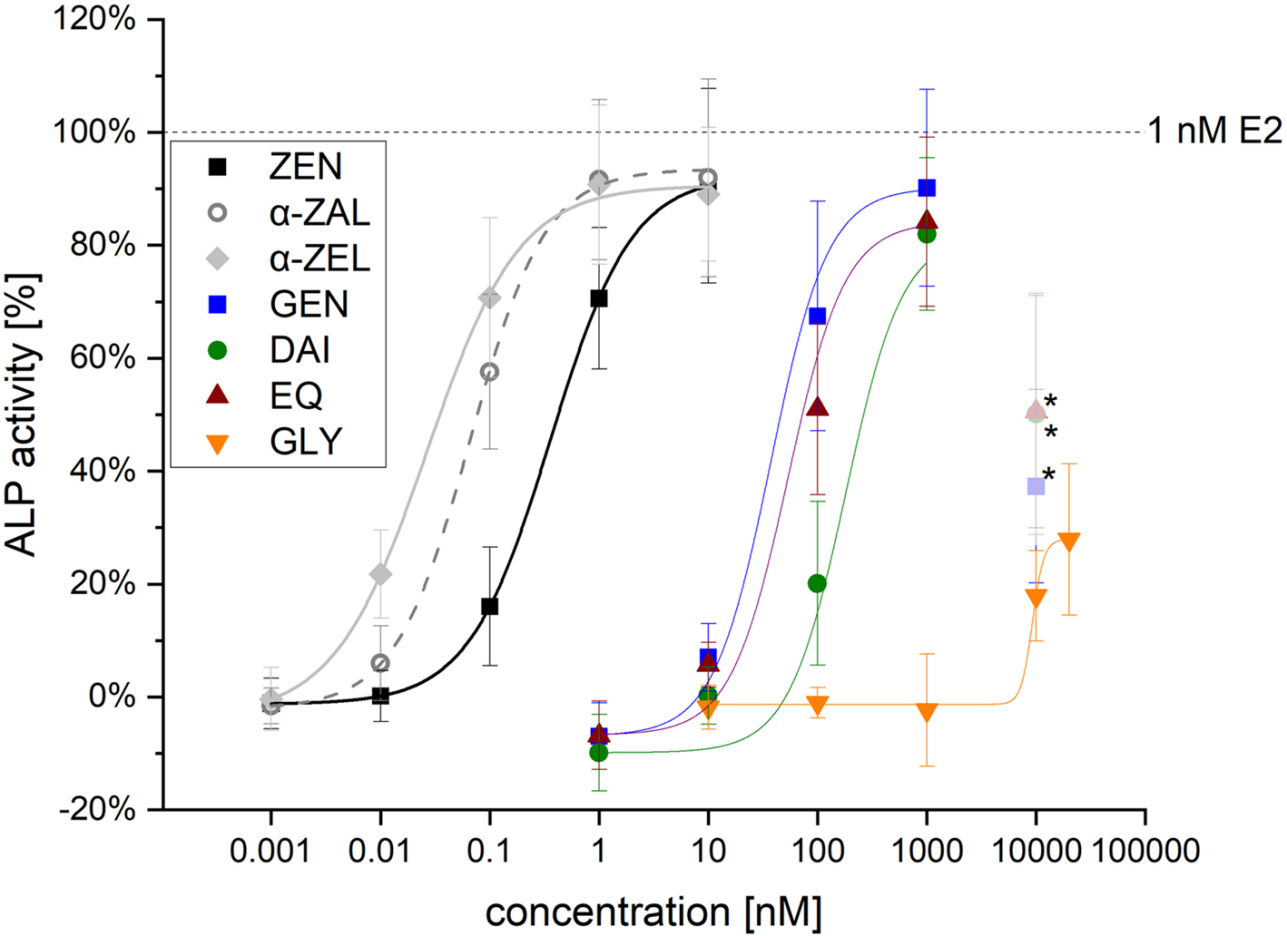
Dose–response curves of zearalenone (ZEN), α-zearalanol (α-ZAL), α-zearalenol (α-ZEL) and the isoflavones genistein (GEN), daidzein (DAI), equol (EQ) and glycitein (GLY). Sigmoidal-dose–response curve fits of estrogen-dependent activation of alkaline phosphatase activity (ALP) in Ishikawa cells after 48 h incubation. Results are depicted as mean ± standard deviation of at least 17 biological replicates (measurements with different cell passages), calculated from the mean value of three technical replicates (repeated measurements with the same cell passage). Outliers after the Nalimov outlier test as well as values marked with * were not included in the sigmoidal-dose-response curve fit. Effects of the solvent control (1% DMSO) and 1 nM E2 as positive control were set to 0 and 100%, respectively

For the mycoestrogens, the most pronounced estrogenic effect was induced by α-ZEL followed by α-ZAL and ZEN with increasing concentrations (Fig. 4), while ZEN-14-S did not induce the ALP activity in the tested concentration range. Furthermore, in a concentration-dependent manner the tested ISF induced an estrogenic response up to a concentration of 1 µM with the highest impact of GEN, followed by EQ and DAI (Fig. 4). At a concentration of 10 µM, a decrease in ALP induction was observed for all three ISF. GLY showed only marginal estrogenic effects and a slight increase in ALP activity starting from 10 µM was observed which further increased at 20 µM.

The highest ALP activity was induced at a concentration of 10 nM by ZEN, α-ZEL and α-ZAL, which was about 90% compared to 1 nM E2. A concentration of 1 µM GEN, DAI and EQ showed the highest ALP induction of 82–90% related to 1 nM E2. In contrast, 20 µM GLY only reached 28 ± 13% of the ALP induction of 1 nM E2.

Sigmoidal dose–response curve fitting was performed for every single substance. By this analysis, the effective concentration that induces a 50% response (EC_50_), a commonly used measure of toxin potency, can be determined. The obtained EC_50_ values for the mycoestrogens were 0.027 ± 0.003 nM (α-ZEL), 0.067 ± 0.004 nM (α-ZAL) and 0.359 ± 0.001 nM (ZEN), and for the ISF 0.037 µM (GEN), 0.054 µM (EQ), 0.181 µM (DAI) and 9.25 µM (GLY). Since only the measured values for the lower four ISF concentrations were used for the EC_50_ calculation, no standard deviation can be provided.

### ALP of combinations

**Fig. 5:**
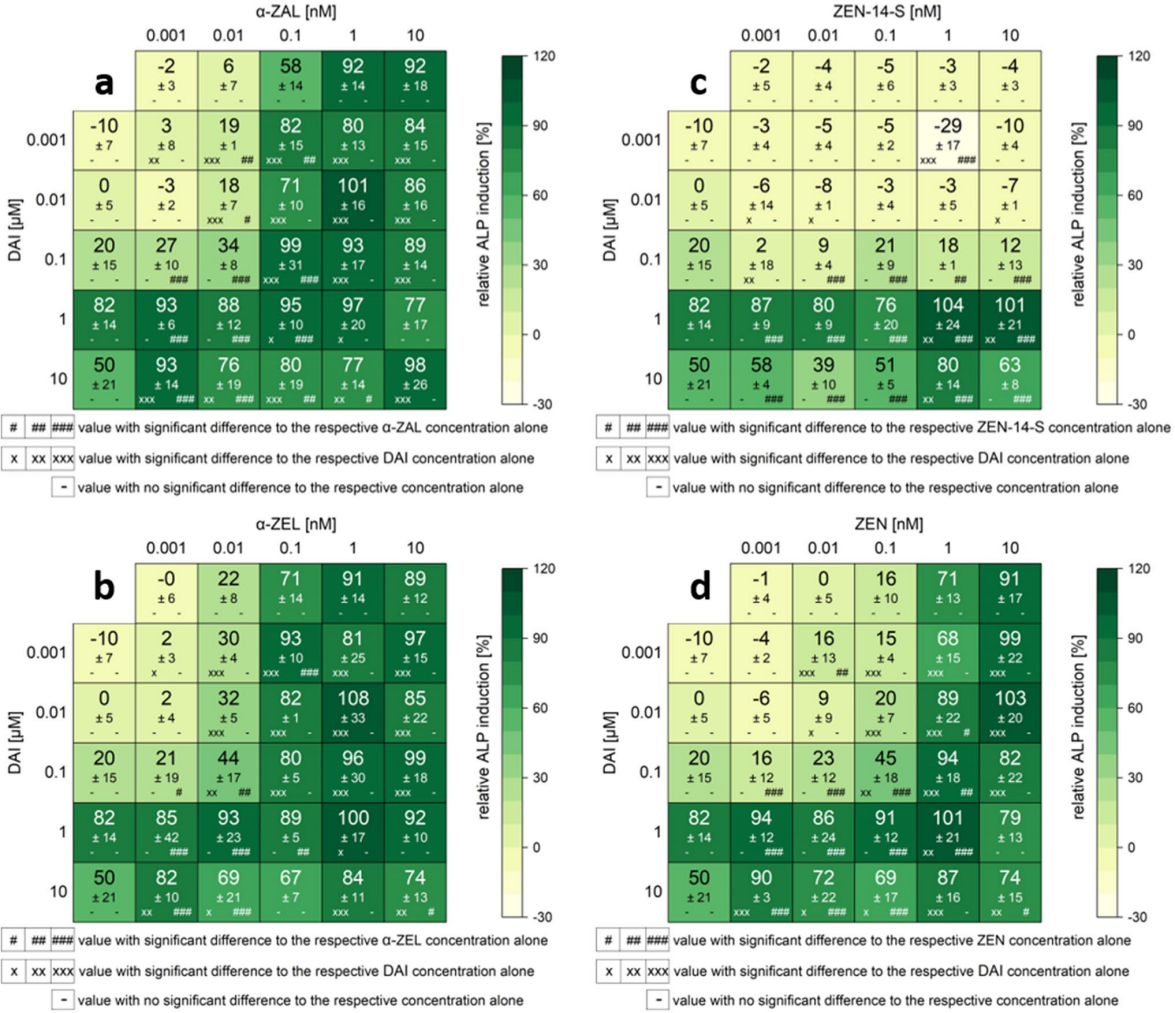
Effects of the combination of mycoestrogens with daidzein (DAI) on the ALP activity. Heatmaps indicating effects of single substances and combinations of α-zearalanol (α-ZAL) (**a**), α-zearalenol (α-ZEL) (**b**), zearalenone-14-sulfate (ZEN-14-S) (**c**) and zearalenone (ZEN) (**d**) with DAI on the alkaline phosphatase (ALP) activity in Ishikawa cells after 48 h incubation. Results are depicted as mean ± standard deviation of at least four biological replicates (measurements with different cell passages), calculated from the mean value of three technical replicates (repeated measurements with the same cell passage). Outliers after the Nalimov outlier test were excluded. Effects of the solvent control (1% DMSO) and 1 nM E2 as positive control were set to 0 and 100%, respectively. The color code indicates the strength of the effects. Normal distribution of data was tested according to Shapiro–Wilk normality test and significance by one-way ANOVA. Significant differences in effects to the respective single substance concentration were indicated with *x* = *p* < 0.05, *xx* = *p* < 0.01 and *xxx* = *p* < 0.001 in the case of DAI and # = *p* < 0.05, ## = *p* < 0.01 and ### = *p* < 0.001 in case of mycoestrogens.“−” corresponds to no significant difference to the respective concentration of the single substance

**Fig. 6:**
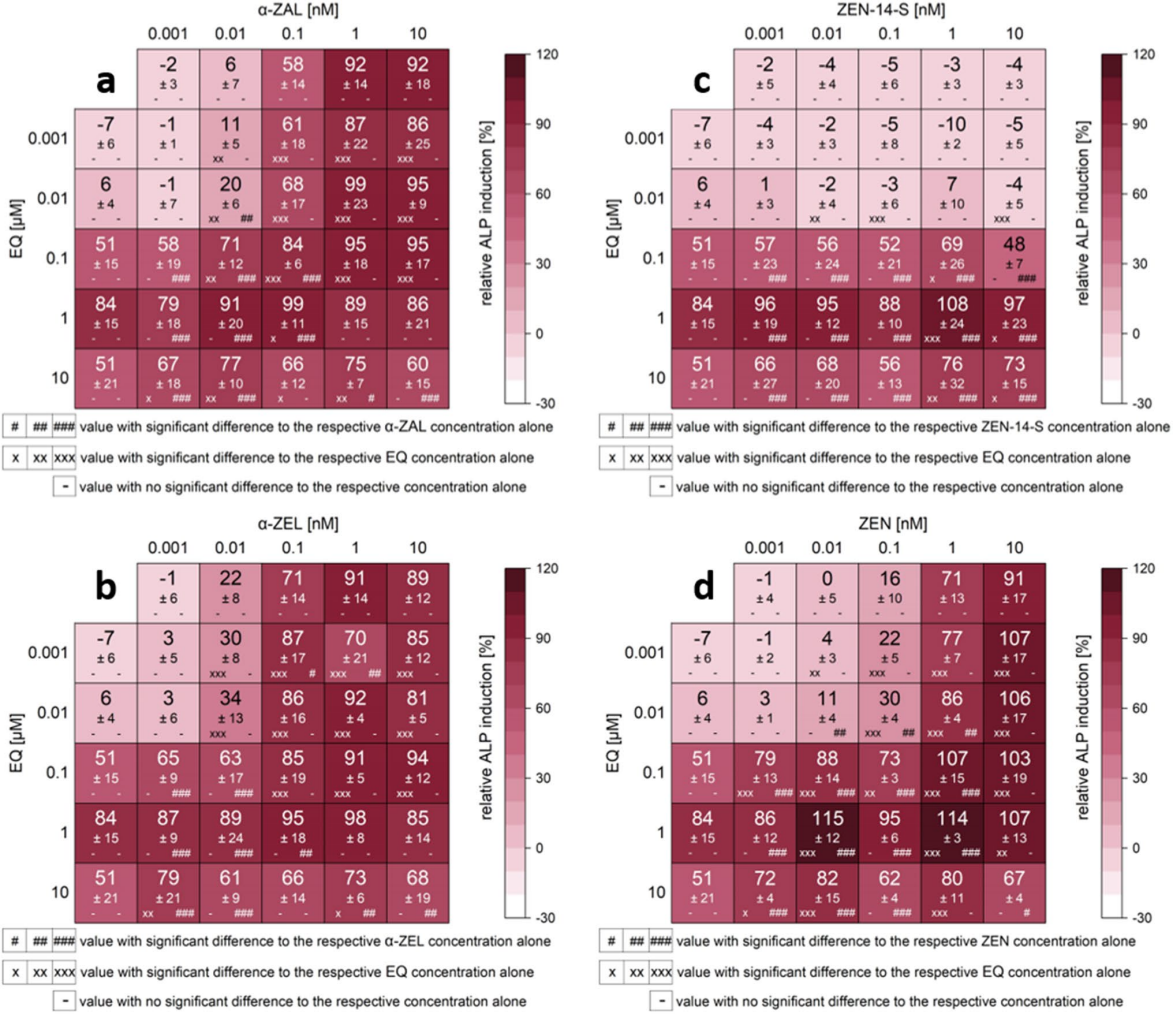
Effects of the combination of mycoestrogens with equol (EQ) on the ALP activity. Heatmaps indicating effects of single substances and combinations of α-zearalanol (α-ZAL) (**a**), α-zearalenol (α-ZEL) (**b**), zearalenone-14-sulfate (ZEN-14-S) (**c**) and zearalenone (ZEN) (**d**) with EQ on the alkaline phosphatase (ALP) activity in Ishikawa cells after 48 h incubation. Results are depicted as mean ± standard deviation of at least four biological replicates (measurements with different cell passages), calculated from the mean value of three technical replicates (repeated measurements with the same cell passage). Outliers after the Nalimov outlier test were excluded. Effects of the solvent control (1% DMSO) and 1 nM E2 as positive control were set to 0 and 100%, respectively. The color code indicates the strength of the effects. Normal distribution of data was tested according to Shapiro–Wilk normality test and significance by one-way ANOVA. Significant differences in effects on the respective single substance concentration were indicated with *x* = *p* < 0.05, *xx* = *p* < 0.01 and *xxx* = *p* < 0.001 in the case of EQ and # = *p* < 0.05, ## = *p* < 0.01 and ### = *p* < 0.001 in case of mycoestrogens. “−”corresponds to no significant difference in the respective concentration of the single substance

**Fig. 7:**
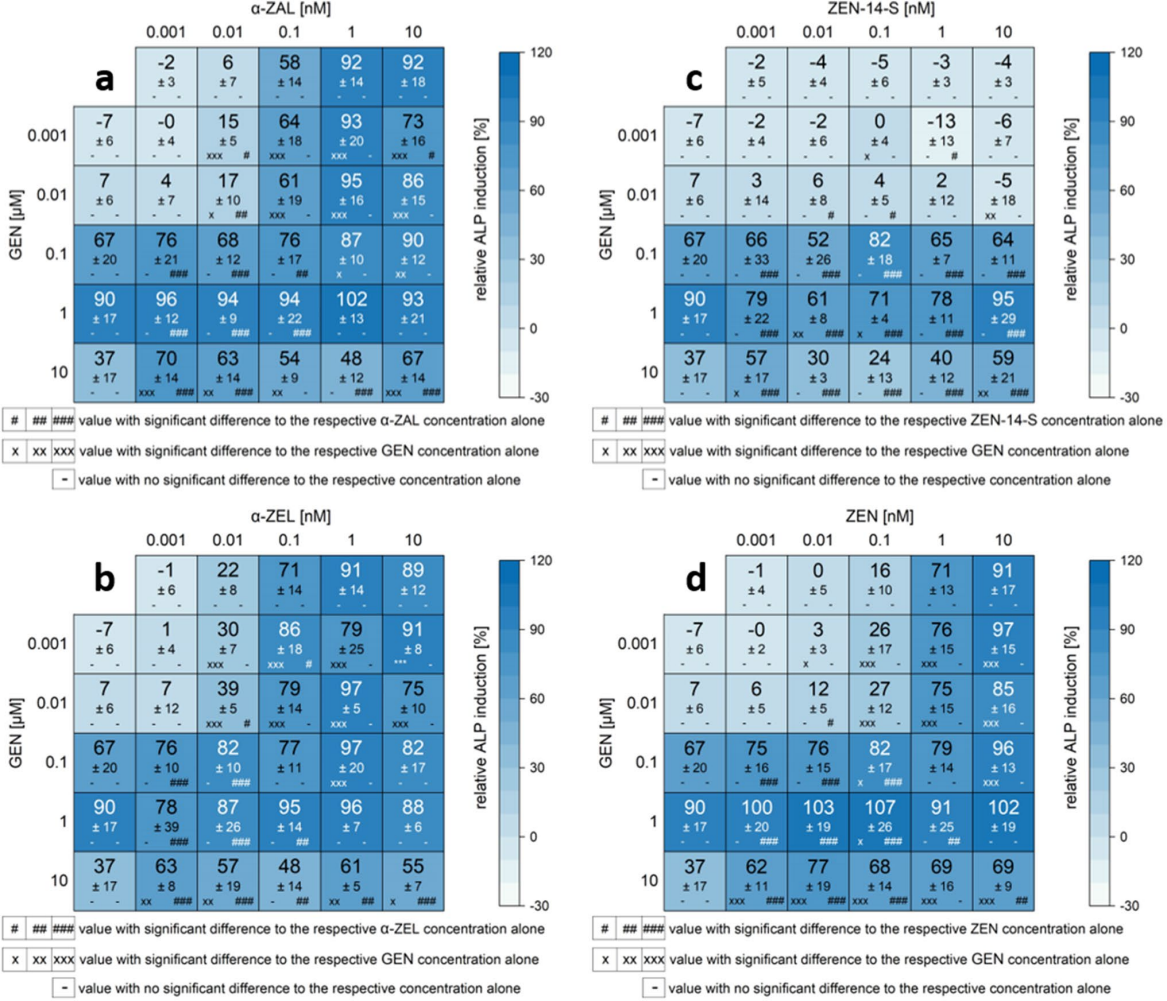
Effects of the combination of mycoestrogens with genistein (GEN) on the ALP activity. Heatmaps indicating effects of single substances and combinations of α-zearalanol (α-ZAL) (**a**), α-zearalenol (α-ZEL) (**b**), zearalenone-14-sulfate (ZEN-14-S) (**c**) and zearalenone (ZEN) (**d**) with GEN on the alkaline phosphatase (ALP) activity in Ishikawa cells after 48 h incubation. Results are depicted as mean ± standard deviation of at least four biological replicates (measurements with different cell passages), calculated from the mean value of three technical replicates (repeated measurements with the same cell passage). Outliers after Nalimov outlier test were excluded. Effects of the solvent control (1% DMSO) and 1 nM E2 as positive control were set to 0 and 100%, respectively. The color code indicates the strength of the effects. Normal distribution of data was tested according to Shapiro–Wilk normality test and significance by one-way ANOVA. Significant differences in effects on the respective single substance concentration were indicated with *x* = *p* < 0.05, *xx* = *p* < 0.01 and *xxx* = *p* < 0.001 in case of GEN and # = *p* < 0.05, ## = *p* < 0.01 and ### = *p* < 0.001 in case of mycoestrogens. “−”corresponds to no significant difference in the respective concentration of the single substance

All measured effects of single substances and combinations are compiled in the heatmaps (Figs. 5, 6, 7, S1). Results are expressed as a percentage of induction, where 0 and 100% represent the values of the solvent control (1% DMSO) and of 1 nM E2, respectively. Significant ALP activation of combinations compared to their respective single substances is indicated by “*x*” in the case of ISF and by “#” for mycoestrogens. The color code of these heatmaps indicates the strength of the effect, which enables a visual interpretation of the results of all tested combinations.

For all data sets, the combinations of ISF with ZEN, α-ZEL and α-ZAL showed similar trends, except for GLY. Effects were most pronounced at low to medium concentrations but decreased at higher doses. ISF concentrations in a range of 0.01–1 µM were found to mediate the most potent effects on the estrogenic activity of mycoestrogens. Except for GLY (Fig. S1), which potentiated the estrogenic effect of low mycoestrogen concentrations at higher levels (10–20 µM). A threshold to enhance the estrogenicity of mycoestrogens was found between 0.01 and 0.1 µM depending on the ISF (Figs. 5, 6, 7).

**Fig. 8:**
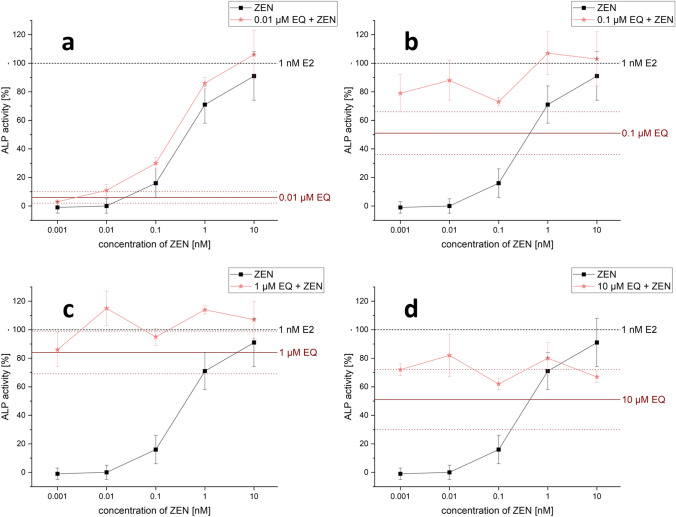
Selected diagrams for the combinatory estrogenic effect of zearalenone (ZEN) and equol (EQ). Illustration of the combinatorial estrogenic effects of ZEN and EQ on the basis of increasing ZEN concentrations and fixed EQ concentrations on the alkaline phosphatase (ALP) activity in Ishikawa cells after 48 h incubation. Results are depicted as mean and standard deviation of at least four biological replicates (measurements with different cell passages), calculated from the mean value of three technical replicates (repeated measurements with the same cell passage). The fixed EQ concentration is displayed as a red line and the corresponding standard deviation as dashed red lines. Results were referred to 1 nM E2 as positive control set to 100% and the solvent control (1% DMSO) set to 0%

In binary mixtures with higher mycoestrogen concentrations (1 and 10 nM), GEN, DAI, and EQ only slightly enhanced the ALP induction compared to the mycoestrogens alone. Some combinations of ISF and mycoestrogens even exceeded the ALP activation of 1 nM E2 (values above 100%). The highest ALP activity for combinations of mycoestrogens with GEN, DAI and EQ was mostly induced at a concentration of 1 µM for the ISF. Overall, the combination of EQ and ZEN showed the most potent estrogenic activities with an ALP induction of 115% (combination of 0.01 nM ZEN + 1 µM EQ) (Fig. 6d). In Fig. 8, selected combinations derived from the heat map of Fig. 6d are shown. The line graphs visualize the strength of the respective EQ concentrations in increasing the ALP activity. Additionally, EQ has the strongest effect to potentiate the estrogenic effect in mixtures with α-ZEL and α-ZAL compared to the other ISF.

The highest concentration of 10 µM ISF leads to a significant reduction in ALP activity by 32–53%. This effect was also seen in mixtures with mycoestrogens, albeit they suppressed the reduction. In total, in lower concentrations than 10 µM, GEN, DAI and EQ showed enhancing estrogenic effects in binary mixtures with mycoestrogens. Mixtures of GLY with mycoestrogens showed different results in the applied system. Concentrations up to 1 µM GLY did not lead to any estrogenic effect. This was also seen for most combinations with mycoestrogens. In the concentration range of 0.01 to 1 µM, GLY did not increase the ALP activity of the mycoestrogens compared to their respective single substances. Only starting from 10 µM, GLY showed estrogenic effects. Interestingly, low levels of mycoestrogens (0.001–0.1 nM) increased the ALP activity. In combination with 1 and 10 nM mycoestrogens, the ALP induction was decreased compared to mycoestrogens alone.

The estrogenic effects of the phase II metabolite of ZEN, namely ZEN-14-S, were different compared to ZEN and its phase I metabolites. No induction of ALP activity was observed in the applied concentrations. In combination with low levels of ISF (0.001–0.1 nM), no stimulatory action was observed. However, higher ISF concentrations (1–10 µM) mixed with ZEN-14-S significantly increased the ALP induction in some cases.

### Cytotoxicity

Cytotoxicity of single substances and combinations was measured with the CTB assay to assess the metabolic activity and the SRB assay to assess the protein content. This was done to exclude the generation of artefacts due to cytotoxicity or induced cell growth.

### ZEN and its metabolites

**Fig. 9:**
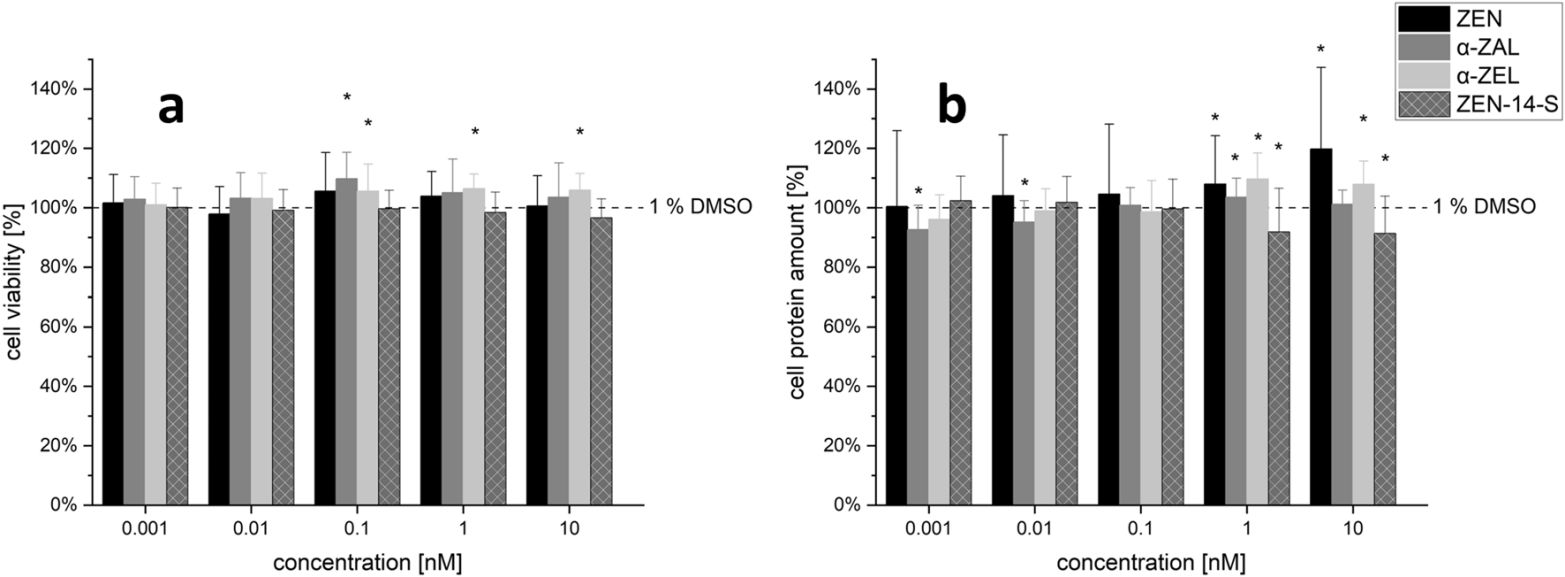
Effects of zearalenone (ZEN), α-zearalanol (α-ZAL), α-zearalenol (α-ZEL) and ZEN-14-sulfate (ZEN-14-S) as single substances on the cytotoxicity. Impact on the cell viability [%] measured by the CellTiter Blue (CTB) assay (**a**) and on the cell protein amount [%] measured by the sulforhodamine B (SRB) assay (**b**) of increasing concentrations of ZEN and its metabolites after 48 h incubation in Ishikawa cells. Values were referred to the solvent control (1% DMSO) set to 100%. Results are depicted as mean ± standard deviation of at least 5 biological replicates (measurements with different cell passages), calculated from the mean value of three technical replicates (repeated measurements with the same cell passage). Outliers after the Nalimov outlier test were excluded. Significant differences of effects between the solvent control and the incubation solutions were calculated by one-sample Student’s *t*-test and significances are indicated with *p < 0.05

No concentration-dependent cytotoxicity could be determined in the tested concentration range of ZEN, α-ZEL, α-ZAL and ZEN-14-S, neither by the CTB nor by the SRB assay (Fig. 9a,  b). However, a slight significant increase in cell viability and protein quantity was determined between test substances and solvent control for some mycoestrogens in higher concentrations. Only α-ZAL in the concentration of 0.001 and 0.01 nM lead to a decreased protein content in the SRB assay. Since α-ZAL showed no decreased protein amount at higher concentrations, this effect was probably not due to cytotoxicity, but due to losses during the processing steps.

### Isoflavones

**Fig. 10:**
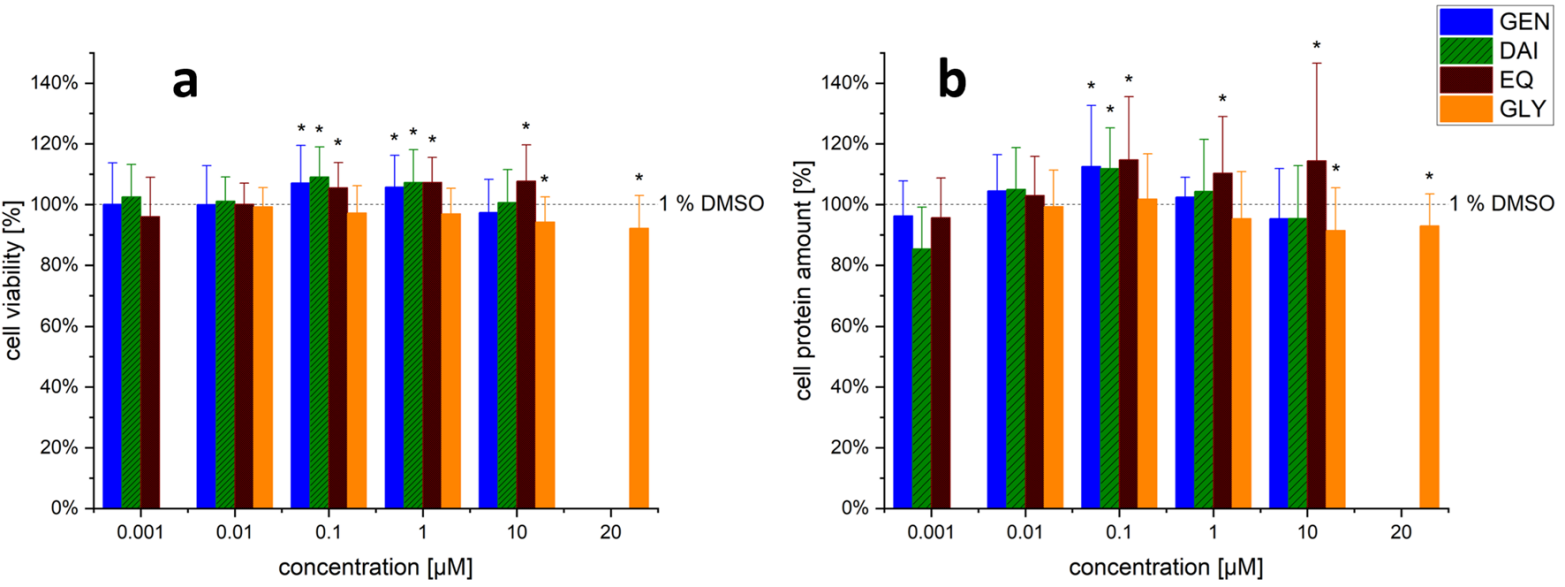
Effects of genistein (GEN), daidzein (DAI), equol (EQ) and glycitein (GLY) on cell viability. Impact on the cell viability [%] measured by the CellTiter Blue (CTB) assay (**a**) and on the cell protein amount [%] measured by the sulforhodamine B (SRB) assay (**b**) of rising concentrations after 48 h incubation in Ishikawa cells. Values were referred to the solvent control (1% DMSO) as 100%. Results are depicted as mean + standard deviation of at least 20 biological replicates (measurements with different cell passages), calculated from the mean value of three technical replicates (repeated measurements with the same cell passage). Outliers after the Nalimov outlier test were excluded. Significant differences in effects between the solvent control and the incubation solutions were calculated by one-sample Student’s *t*-test. Significances are indicated with *(*p* < 0.05). According to its low estrogenicity, GLY was tested in one higher concentration (20 µM) instead of the lowest concentration of 0.001 µM

No significant decrease in metabolic activity (Fig. 10a) or in the cell protein amount (Fig. 10b) was observed for GEN, DAI and EQ. In contrast GEN, DAI and EQ showed a significant tendency in increasing the metabolic activity (Fig. 10a) and cell protein amount (Fig. 10b) at certain concentrations. Concentrations of 10 and 20 µM GLY lead to a slight significant decrease in cell viability and cell protein amount.

### Cytotoxicity of combinations

**Fig. 11:**
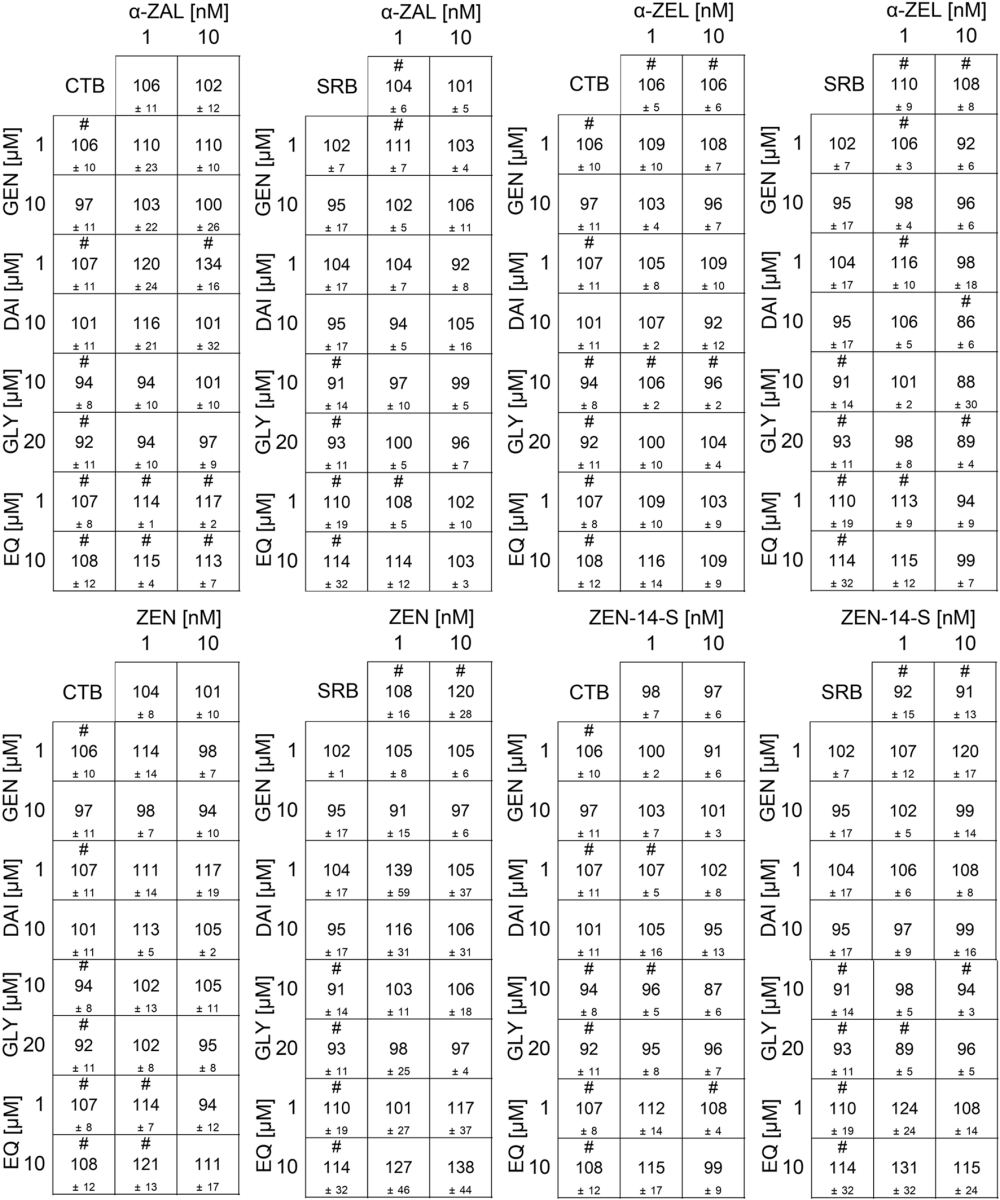
Effects of the combination of mycoestrogens (zearalenone (ZEN), α-zearalanol (α-ZAL), α-zearalenol (α-ZEL) and ZEN-14-sulfate (ZEN-14-S)) with the isoflavones (genistein (GEN), daidzein (DAI), equol (EQ) and glycitein (GLY)) on the cytotoxicity. Impact on the cell viability [%] measured by the CellTiter Blue (CTB) assay and on the cell protein amount [%] measured by the sulforhodamine B (SRB) assay of single substances and combinations of the highest concentrations of ZEN and its metabolites (1 nM and 10 nM) with the highest concentrations of isoflavones (1 µM and 10 µM for GEN, DAI, and EQ or 10 µM and 20 µM for GLY) after 48 h in Ishikawa cells. Values were referred to the solvent control (1% DMSO) as 100%. Results are depicted as mean ± standard deviation of at least four biological replicates (measurements with different cell passages), calculated from the mean value of three technical replicates (repeated measurements with the same cell passage). Outliers after the Nalimov outlier test were excluded. Significant differences in effects between the solvent control and the incubation solutions were calculated by one-sample Student’s *t*-test. Significances are indicated with # (*p* < 0.05)

As shown in Fig. 11, the metabolic activity (CTB assay) and the protein content (SRB assay) of the two highest concentrated combinations between ISF and ZEN and its metabolites are shown exemplarily. No significant decrease in metabolic activity or protein content was induced. However, for some combinations a significant increase in the two applied systems was generated. Furthermore, cytotoxic effects were also assessed in lower concentrations (Supplement Information Figs. S2–S5).

### Combination Index (CI)

**Fig. 12:**
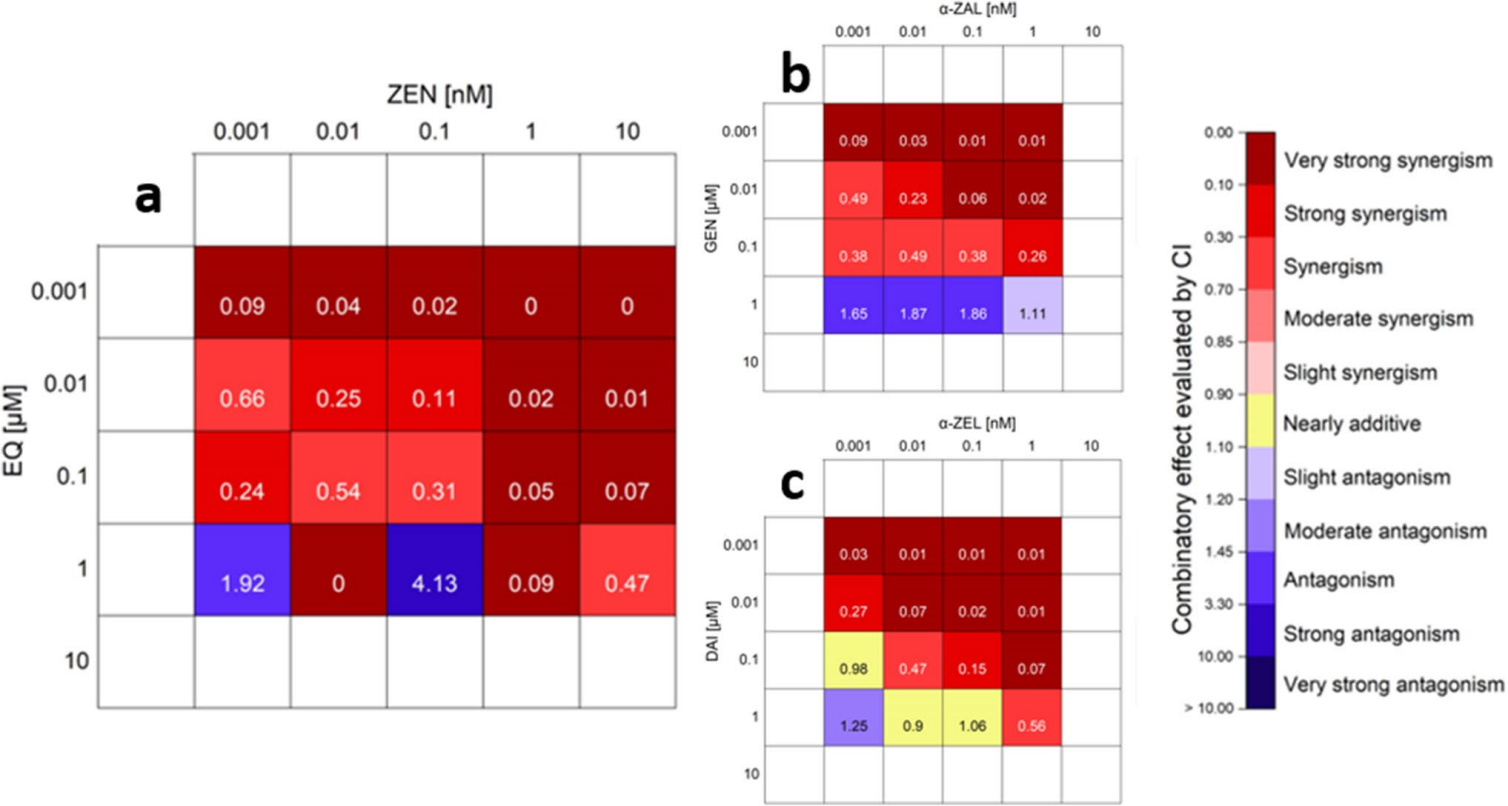
Combination index (CI) of selected combinations of mycoestrogens and isoflavones. Heatmaps indicating the CI of the combination zearalenone (ZEN) with equol (EQ) (**a**), α-zearalanol (α-ZAL) with genistein (GEN) (**b**) and α-zearalenol (α-ZEL) with daidzein (DAI) (**c**) of the estrogenic action measured by alkaline phosphatase (ALP) activity and calculated according to Chou (Chou 2006). The CI was only calculated for the measured values which fulfilled the requirements for calculation. The color code indicates the combinatory effect of the mixture. Red corresponds to values < 0.9 and therefore synergism, yellow for CI between 0.9 and 1.1 for nearly additive effects and blue for antagonism with CI values of > 1.1. In addition, the statistical analysis of the measured dataset obtained from the ALP measurements is present

Some requirements have to be fulfilled for the assessment of the CI. Therefore, no CI calculation was possible for ZEN-14-S and GLY as they had no or only a minor impact on the ALP activity at the applied concentrations. Only the CI values of combinations between GEN, DAI and EQ together with ZEN, α-ZEL and α-ZAL were evaluated. Furthermore, only values between zero and one can be considered. As in some cases, the ALP inductions were lower than 0% or higher than 100%, the effects had to be recalculated and related to -26% and 115% which corresponds to the lowest and highest ALP activity of all single substances and combinations thereof. Additionally, only values which are in the linear range of the dose–response curve may be considered. Thus, 10 µM GEN, DAI and EQ and 10 nM α-ZEL and α-ZAL as single substances were not considered for the CI calculation. We calculated the CI for the combinations α-ZEL with DAI, ZEN with EQ and α-ZAL with GEN to provide a representative for every single substance (see Fig. 12).

For all combinations, the incubation of 0.001 µM ISF with various mycoestrogen concentrations had a very strong synergistic effect on the ALP induction. At ISF concentrations of 0.01 and 0.1 µM, the combinatory effects were strongly synergistic or synergistic. However, at an ISF concentration of 1 µM in combinations with various mycoestrogen concentrations, the combinatory effects varied. For the mixtures between α-ZEL with DAI (1 µM), respectively, the CI values indicate an additive or even antagonistic effect, whereas the combination of ZEN and EQ (1 µM) showed strong to very strong synergism or antagonism.

## Discussion

Consumers are exposed to a wide range of undesirable and partly toxic compounds at the same time. Therefore, not only the individual effect of one substance should be evaluated, but also an interaction with other toxins or bioactive agents should be considered to provide a reliable risk assessment. Previous studies have shown that xenoestrogens can potentiate their toxic effect in vitro (Vejdovszky et al. 2017a, b)*.* As recently reviewed, mycoestrogens and phytoestrogens co-occur together frequently in various animal feed commodities (Grgic et al. 2021). We decided to investigate the estrogenic effect of the three major ISF (GEN, DAI and GLY) found in soy and the gut microbial metabolite of DAI EQ, as soy is one of the most frequently used protein sources in animal feed. On the other hand, corn is the major carbohydrate additive in animal feed. One of the most common mycotoxins found in corn is ZEN with an incidence of 81% (Munkvold et al. 2018). ISF and ZEN are known to exhibit estrogenic properties. However, as ISF occur in higher concentrations and 1000 times higher concentrations are required to induce estrogenic stimuli compared to ZEN and its metabolites, we investigated the combinatory effects in concentration ratios of 1:1—1000:1 (ISF:mycoestrogen) in steps with a factor of 10 in between. This demonstrates the importance to assess their combinatory effects especially when the same mechanism is addressed. Therefore, our aim was to investigate the potential of ISF combined with ZEN and its metabolites on a possible increase of estrogenic effects in vitro.

Measurements of single mycotoxins (Fig. 4) confirmed previous reports that α-ZEL is the most potent estrogen followed by α-ZAL and the parent compound ZEN determined in human MCF-7 (Molina-Molina et al. 2014) and Ishikawa cells (Vejdovszky et al. 2017a; Mendez-Catala 2020). In contrast, ZEN-14-S seems to possess no estrogenic potential due to conjugation. This loss of estrogenic activity after the conjugation at C14 of ZEN was already determined for ZEN-14-glucose in silico experiments (Dellafiora et al. 2017). Under the applied conditions, ZEN-14-S did not seem to deconjugate to ZEN as no increase in ALP activity was observed.

ZEN is able to induce cytotoxic effects at very high concentrations (39.3–78.5 mM; 39.7 ± 9.6 µM) in K 562 (Reubel et al. 1987) and HepG2 cells, respectively (Marin et al. 2019). However, at the applied concentrations of this study (0.001 to 10 nM), no concentration-dependent cytotoxicity could be determined for ZEN, α-ZEL, α-ZAL and ZEN-14-S by assessing the metabolic activity or the protein content in the Ishikawa cells (Fig. 9). On the contrary, some concentrations lead to significant increases of the measured parameters. It can be hypothesized that ZEN or its metabolites may trigger mitochondrial swelling and possibly lead to the formation of megamitochondria which could at least partially lead to increased metabolic activity. This effect has already been hypothesized for deoxynivalenol (Krishnaswamy et al. 2010; Springler et al. 2017). This result indicates that mycoestrogens might have proliferative effects, although the CTB and SRB were only performed to investigate the cytotoxic effect to rule out that a reduced ALP activity is caused by cytotoxicity. Longer incubations than the applied 48 h would have been required to clearly assess proliferative effects, but this is not the focus of the present article.

Especially in soy-containing products and finished feed, ISF usually occur in higher (100–1000 times) concentrations compared to mycoestrogens (Lee et al. 2003; Grgic et al. 2021). Therefore, ISF are consumed in higher amounts by mammals with plasma concentrations reaching up to a low two-digit µM range (Vitale et al. 2013). Thus, a wide range up to a concentration of 20 µM ISF was tested to assess the estrogenic potency. As seen in Fig. 4, the induction of ALP expression by GEN, DAI and EQ increased concentration-dependently, except for the highest tested concentration (10 µM). In case of GLY only the two highest applied concentrations (10 and 20 µM) enhanced ALP expression and only to a limited percentage compared to 1 nM E2 (by 18 ± 8% and 28 ± 13%, respectively). Compared to ZEN and its metabolites, 100 to 1000 times higher concentrations of ISF were required to reach the same estrogenic impact. Taken together, in Ishikawa cells the estrogenic potential of ISF can be ranked as GEN > EQ > DAI >  >  > GLY.

Other studies indicate that EQ has the highest affinity to bind to ER using human estrogen receptor (hER) transfected *Saccharomyces cerevisiae* and MCF-7 cells (Morito et al. 2001; Mortensen et al. 2009). However, the ALP induction in Ishikawa cells after GEN and EQ incubation hardly differ and estrogenic effects may vary between cell lines, applied assay system and measured endpoint. Furthermore, GEN and EQ are ranked by the United Kingdom Committee on Toxicity of Chemicals in Food, Consumer Products and the Environment (COT) to possess the highest estrogenic potential of all ISF (Committee on Toxicity of Chemicals in Food, Consumer Products and the Environment 2011).

In the case of GEN, DAI and EQ the highest applied concentration (10 µM) lead to a minor induction of the ALP compared to 1 µM. This decreased ALP activity for 10 µM ISF cannot be fully explained by a slight cytotoxic effect but might arise from an overlay of different cellular mechanisms. In the case of GEN, a spectrum of cellular mechanisms in different concentration ranges have been reported, including inhibition of protein kinases (Chang and Geahlen 1992; Kurzer and Xu 1997). Phosphorylation steps represent important signals in the ER pathway, potentiating ER genomic signaling activity on gene transcription (Arpino et al. 2008). Therefore, it might be speculated that inhibitory effects on respective kinases are involved in the apparent suppression of estrogenic response in the highest applied concentration of GEN, DAI and EQ.

A further possible explanation could be a suppression of estrogen receptor expression. This has already been shown in in vivo experiments, where high doses of ISF were able to decrease *ESR1* (gene encoding estrogen receptor α) mRNA levels in rat uterus (Cotroneo et al. 2001). Furthermore, higher ISF concentrations are able to induce the transcription of cytochrome P450 family 1 subfamily B member 1 (*CYP1B1*), which in turn leads to increased metabolism of xenoestrogens and might be another explanation for the reduced estrogenicity at the highest concentration (Satih et al. 2010; Wei et al. 2015).

Phase II metabolism of ISF may play a further role in the reduced estrogenic activity. In humans mainly sulfoglucuronides and diglucuronides circulate in the human body (Hosoda et al. 2011; Soukup et al. 2016). This was also observed for ISF in endothelial cells where the glucuronide and sulfate conjugates are predominant (Toro-Funes et al. 2015). This conjugation is considered to detoxify the isoflavones, which might lead to reduced estrogenic effects (Setchell et al. 1987). However, there is also data that indicates that phase II metabolites of ISF are still biologically active, albeit to a much lower extent than their parent compounds (Hüser et al. 2018; Pugazhendhi et al., 2008). This could be a further possible explanation for the reduced estrogenic activity of ISF at the highest concentration.

Regarding combinatory effects between mycoestrogens and ISF, the results demonstrated that for most combinations higher ALP inductions were achieved compared to the respective single substances (Figs. 5, 6, 7). Taken together, the results of the CI calculation indicate synergistic estrogenic effects between ZEN, α-ZEL, α-ZAL and GEN, DAI, EQ. These effects were most pronounced at lower concentrations of mycoestrogens and ISF. With increasing ISF concentrations (10 µM), the interaction shifted towards additive or even antagonistic effects (Fig. 12). At this ISF concentration (10 µM), in some cases, the addition of medium to high concentrations of mycoestrogens led to a lower induction of ALP activity compared to the respective single substances. A possible explanation for this effect might be that ISF in these high concentrations are saturating and binding to both ER and therefore, the mycoestrogen is not able to exert its estrogenic potential (Nikov et al. 2000). In general, the combinations of the parent mycotoxin ZEN with ISF showed the most potent estrogenic stimulus, which was reflected by the respective CI (Fig. 12). This was unexpected as the phase I metabolites α-ZEL and α-ZAL as single compounds had stronger estrogenic effects (Fig. 4), suggesting that their estrogenic impact might arise also in combination. The most prominent enhancing effect was observed with the combination of ZEN and EQ where an ALP induction of up to 115 ± 12% was reached (Fig. 6d, 12a). However, α-ZEL and α-ZAL in mixtures with ISF also increased the ALP expression for some combinations significantly (Figs. 5, 6, 7). Out of all ISF, GLY showed the lowest estrogenic potential as a single substance, which was also seen in combinations with mycoestrogens (Fig. S1).

We hypothesized that the enhanced estrogenic effects of combinations between phyto- and mycoestrogens are based on the interaction of the respective substances with either ERα or ERβ, which are both expressed by Ishikawa cells. While ZEN and its metabolites have a stronger affinity to bind to both ER, ISF preferably interact with ERβ (Nikov et al. 2000; Setchell et al. 2005; Takemura et al. 2007). This has already been elucidated for ZEN, GEN and EQ for the human ERα and ERβ. ZEN shows a similar relative binding affinity (RBA) to ERα and ERβ, which is 8 and 11, respectively. In contrast, the RBA for GEN to bind to ERα and ERβ is dissimilar. Its RBA to ERβ is significantly higher (RBA = 31) compared to binding to ERα (RBA = 1). This observation was also seen for EQ, where its affinity increases from 0.3 for ERα to 3 for ERβ.

ZEN-14-S as a phase II metabolite is classified as a masked mycotoxin and might be hydrolyzed to ZEN after entering the colon through the enterohepatic cycle. Furthermore, there were no current studies on the estrogenic potential of ZEN-14-S. In contrast to the phase I metabolites, ZEN-14-S had no effects on ALP activity as a single substance in the applied system, so enhancing estrogenic effects in combinations with ISF were not expected. Only in some cases, high ISF concentrations lead to a slightly increased trend in ALP activity. The conjugation of ZEN with glucuronides has already been described as a detoxification process (Dellafiora et al. 2017). The sulfate conjugate showed no estrogenic properties neither as a single substance nor in combination with ISF and is therefore considered as a detoxifying metabolite.

For GEN, DAI and EQ as single compounds, the onset of estrogenic response is observed at a threshold value between 0.01 and 0.1 µM. Several studies indicated that in populations with high soy consumption the ISF blood plasma levels can reach 0.1 to 0.9 µM (Gooderham et al. 1996; Verkasalo et al. 2001). In this concentration range, GEN, DAI and EQ already mediate estrogenic effects. However, in mixtures with mycoestrogens these low concentrations are able to potentiate the estrogenic effects. In farm animals even higher ISF plasma concentrations can be detected between 1 and 10 µM (Grgic et al. 2021). In these high concentrations combined with mycoestrogens even higher ALP activities were induced compared to 1 nM E2. In contrast, in tissue and cells where only one ER is predominant, like it is the case for estrogen-sensitive breast cancer cells (ERα) no enhanced estrogenic effects are expected. On the contrary, high ISF concentrations (> 1 µM) could suppress estrogenic effects which are induced by mycoestrogens. Our study demonstrates the interactive effects of phyto- and mycoestrogens, which would require verification in in vivo studies to clarify the consumer´s risk. However, it is necessary that not only the toxicity of single substances is considered for risk assessments, but also combinatory effects of realistic uptake scenarios to ensure consumer’s safety. This in fact would indicate that the maximal tolerable amount of ZEN in various food and feed stuff should be reconsidered and subsequently adopted, as synergistic effects may increase the hazard. Furthermore, the risk assessment for phytoestrogens should be discussed with regard to the high exposure from feed and resulting possible negative effects affecting the reproductive system of certain farm animals.

## Supplementary Information

Below is the link to the electronic supplementary material.

Supplementary file1 (PDF 2216 KB)
