# Supplementary material for: Estrogenic in vitro evaluation of zearalenone and its phase I and II metabolites in combination with soy isoflavones
Source: Arch Toxicol. 2022 Aug 20;96(12):3385–402. doi: 10.1007/s00204-022-03358-3 (PMC9584851; doi:10.1007/s00204-022-03358-3)
Supplement: Supplementary file 1 — Supplementary file1 (PDF 2216 KB) [file 204_2022_3358_MOESM1_ESM.pdf]

Supplementary information of

Estrogenic *in vitro* evaluation of zearalenone and its phase I and II metabolites in combination with soy isoflavones

Dino Grgic[1,2], Andrea Betschler[1], Rebeka Früholz[1], Barbara Novak[3], Elisabeth Varga[1,*], Doris Marko[1,*]

[1] Department of Food Chemistry and Toxicology, Faculty of Chemistry, University of Vienna, Währinger Str. 38-40, 1090 Vienna, Austria

[2] University of Vienna, Doctoral School in Chemistry, Währinger Str. 38-40, 1090 Vienna, Austria

[3] DSM - BIOMIN Research Center, Technopark 1, 3430 Tulln, Austria

* corresponding authors: Elisabeth Varga, elisabeth.varga@univie.ac.at, +43-1-4277-70811
Doris Marko, doris.marko@univie.ac.at, +43-1-4277-70800

Alkaline phosphatase activity of mycoestrogens together with GLY

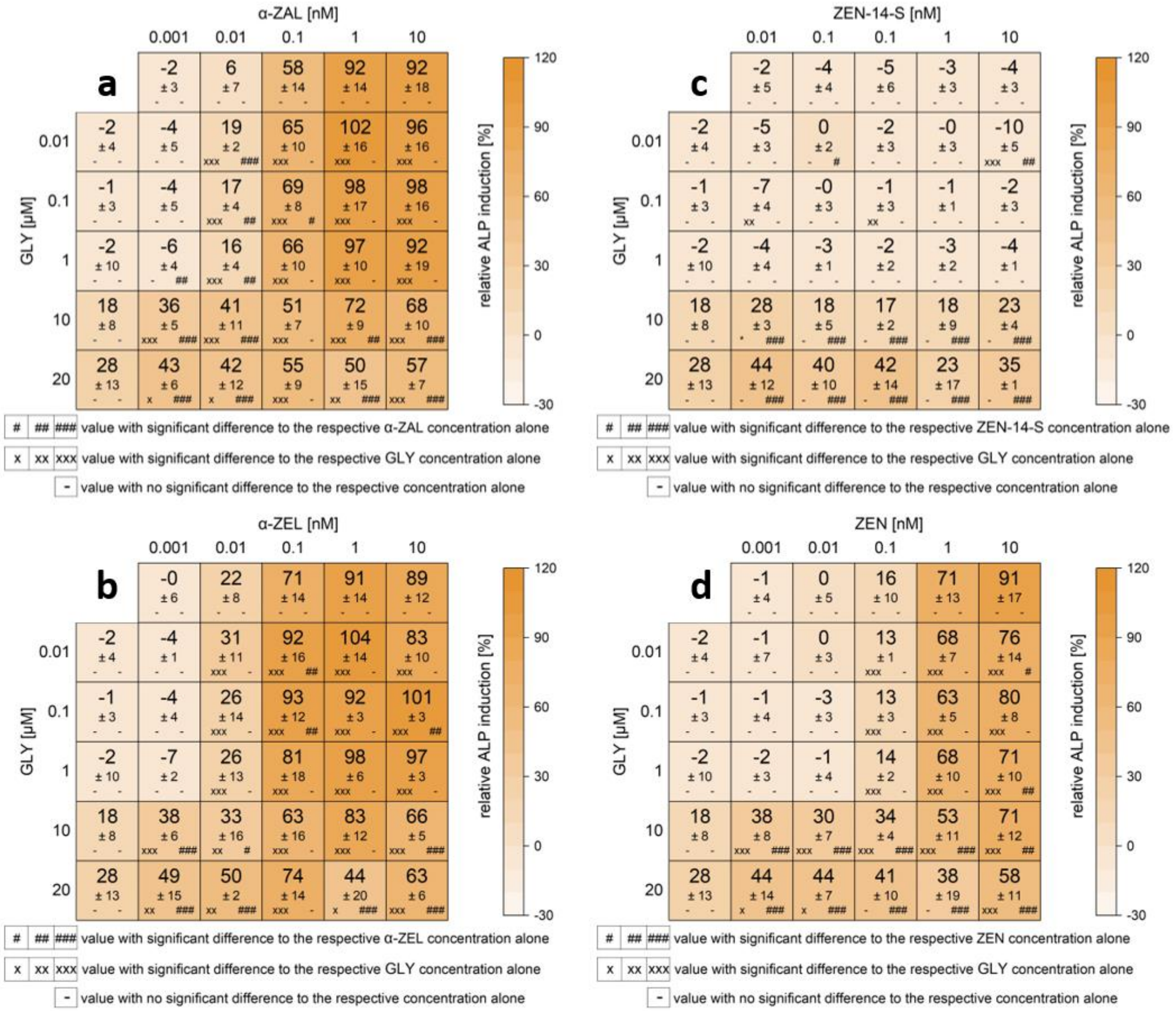

Fig. S1 Effects of the combination of mycoestrogens with glycitein (GLY) on the ALP activity

Heatmaps indicating effects of single substances and combinations of α-zearalanol (α-ZAL) (a), α-zearalenol (α-ZEL) (b), zearalenone-14-sulfate (ZEN-14-S) (c) and zearalenone (ZEN) (d) with GLY on the alkaline phosphatase (ALP) activity in Ishikawa cells after 48 h incubation. Results are depicted as mean ± standard deviation of at least four biological replicates (measurements with different cell passages), calculated from the mean value of three technical replicates (repeated measurements with the same cell passage). Outliers after Nalimov outlier test were excluded. Effects of the solvent control (1 % DMSO) and 1 nM E2 as positive control were set to 0 and 100 %, respectively. The color code indicates the strength of the effects. Normal distribution of data was tested according to Shapiro-Wilk normality test and significance by one-way ANOVA. Significant differences of effects to the respective single substance concentration were indicated with x = $p < 0.05$, xx = $p < 0.01$ and xxx = $p < 0.001$ in case of GLY and # = $p < 0.05$, ## = $p < 0.01$ and ### = $p < 0.001$ in case of mycoestrogens. "-" corresponds to no significant difference to the respective concentration of the single substance

Cytotoxicity of combinations

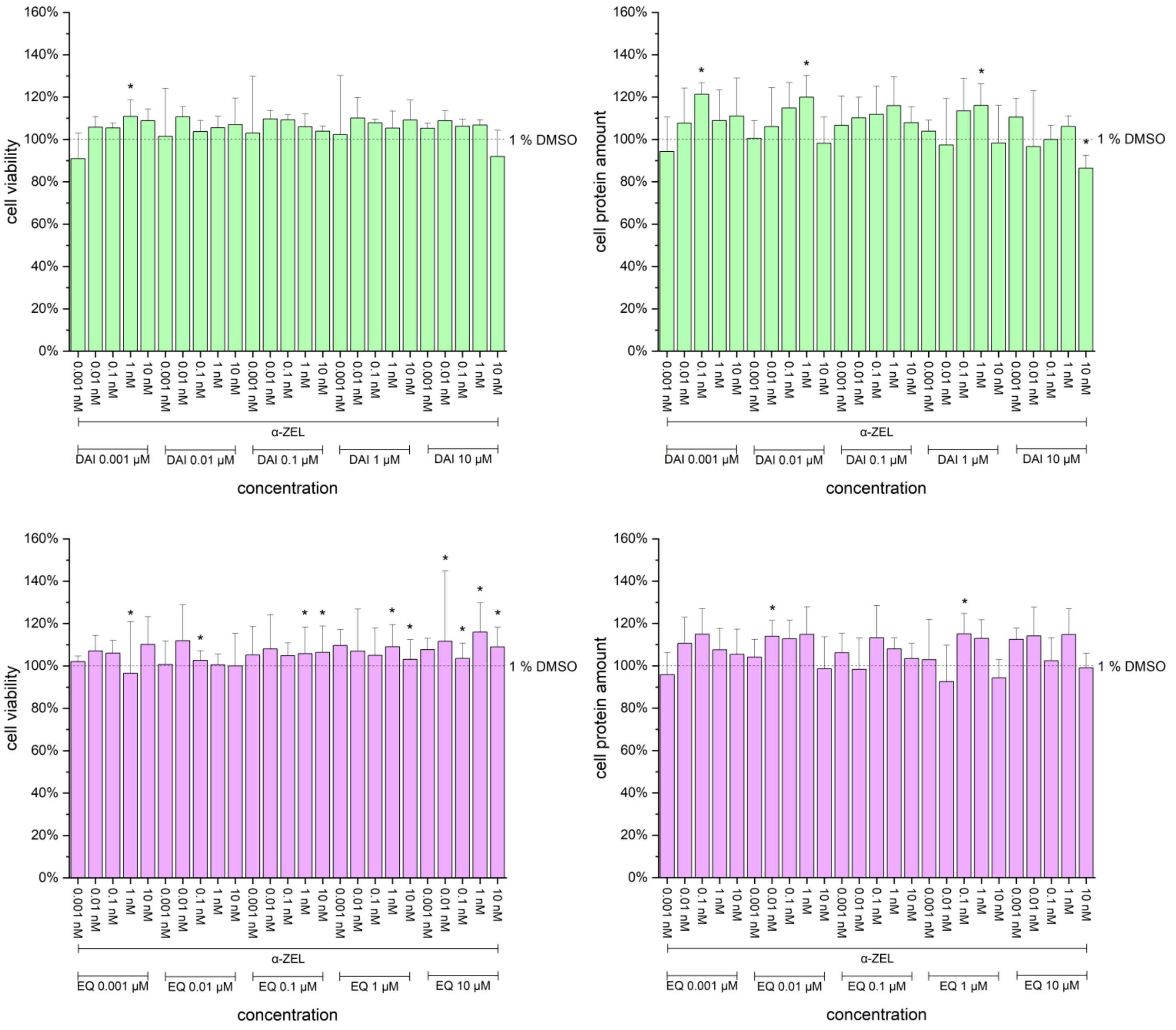

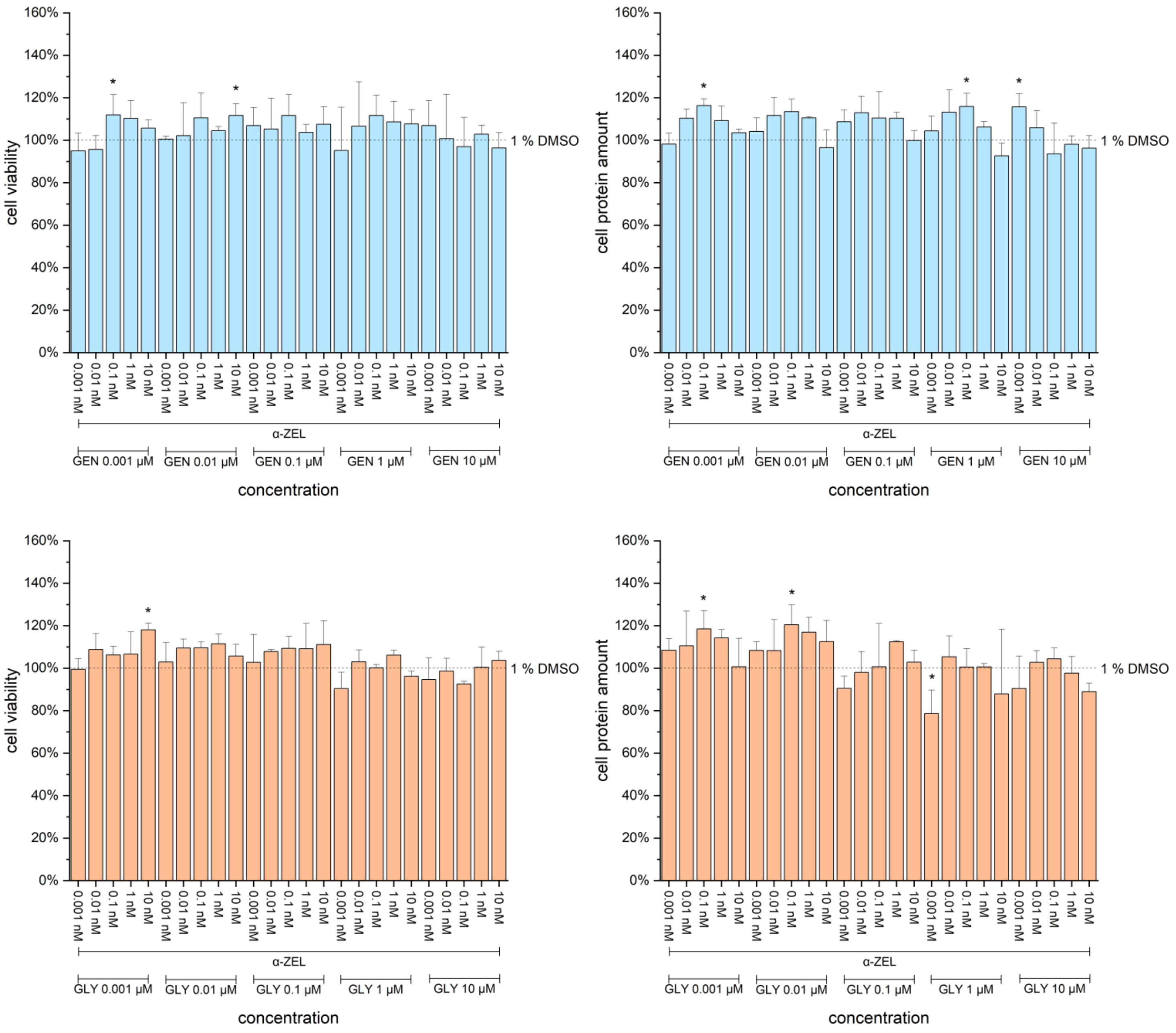

Fig. S2 Effects of the combination of α-zearalenol (α-ZEL) with isoflavone on the cell viability

Impact on the cell viability [%] measured by the CellTiter Blue (CTB) assay (**left**) and on the cell protein amount [%] measured by the sulforhodamine B (SRB) assay (**right**) of different combinations and concentrations after 48 h incubation in Ishikawa cells. Values were referred to the solvent control (1 % DMSO) as 100 %. Results are depicted as mean + standard deviation of at least four biological replicates, calculated from the mean value of three technical replicates. Outliers after Nalimov outlier test were excluded. Significant differences of effects between the solvent control and the incubation solutions were calculated by one-sample Student's *t*-test. Significances are indicated with * ($p < 0.05$). According to its low estrogenicity, GLY was tested in one higher concentration (20 µM) instead of the lowest concentration of 0.001 µM.

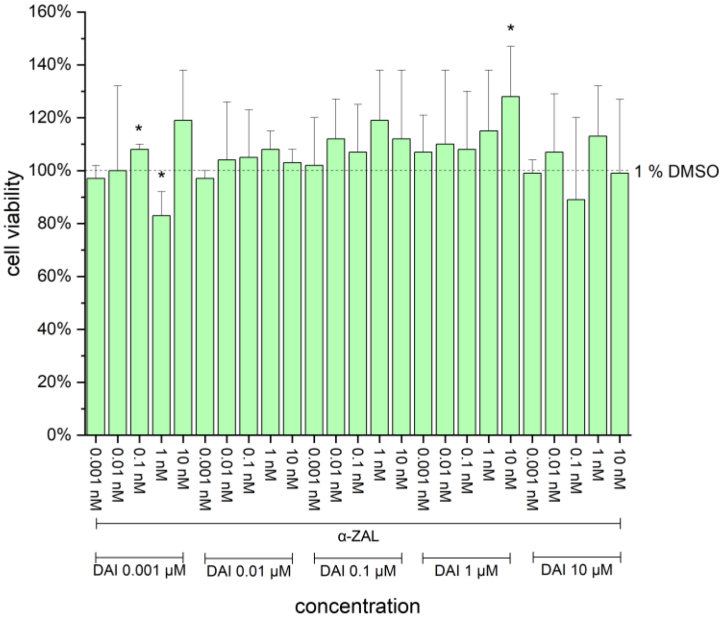
160%
140%
120%
100%
80%
60%
40%
20%
0%
cell viability
1 % DMSO
0.001 nM
0.01 nM
0.1 nM
1 nM
10 nM
α-ZAL
DAI 0.001 µM
DAI 0.01 µM
DAI 0.1 µM
DAI 1 µM
DAI 10 µM
concentration

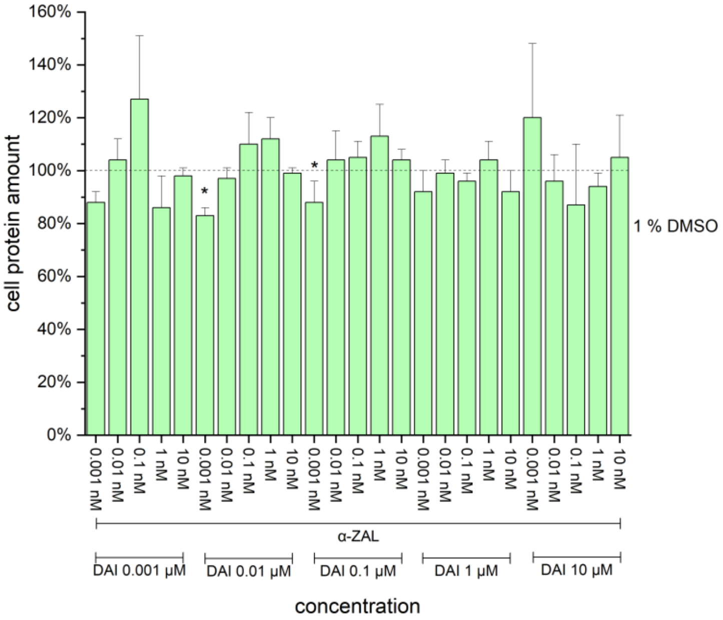
160%
140%
120%
100%
80%
60%
40%
20%
0%
cell protein amount
1 % DMSO
0.001 nM
0.01 nM
0.1 nM
1 nM
10 nM
α-ZAL
DAI 0.001 µM
DAI 0.01 µM
DAI 0.1 µM
DAI 1 µM
DAI 10 µM
concentration

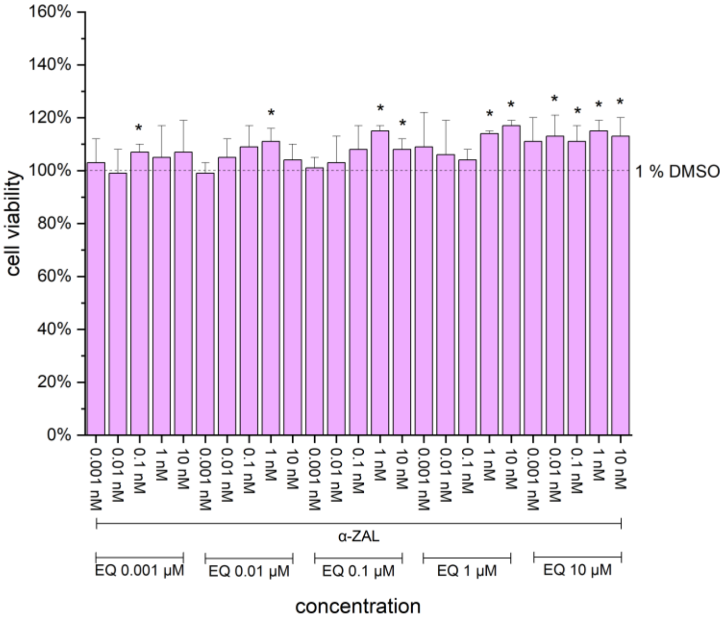
160%
140%
120%
100%
80%
60%
40%
20%
0%
cell viability
1 % DMSO
0.001 nM
0.01 nM
0.1 nM
1 nM
10 nM
α-ZAL
EQ 0.001 µM
EQ 0.01 µM
EQ 0.1 µM
EQ 1 µM
EQ 10 µM
concentration

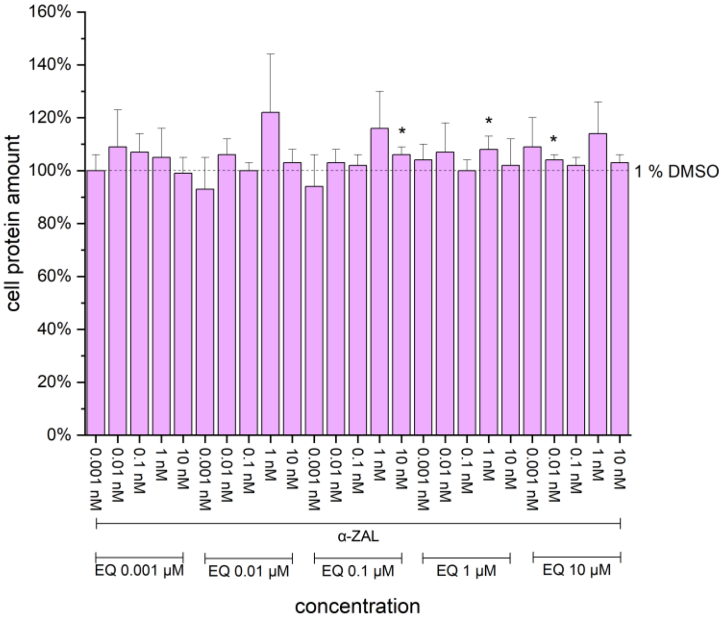
160%
140%
120%
100%
80%
60%
40%
20%
0%
cell protein amount
1 % DMSO
0.001 nM
0.01 nM
0.1 nM
1 nM
10 nM
α-ZAL
EQ 0.001 µM
EQ 0.01 µM
EQ 0.1 µM
EQ 1 µM
EQ 10 µM
concentration

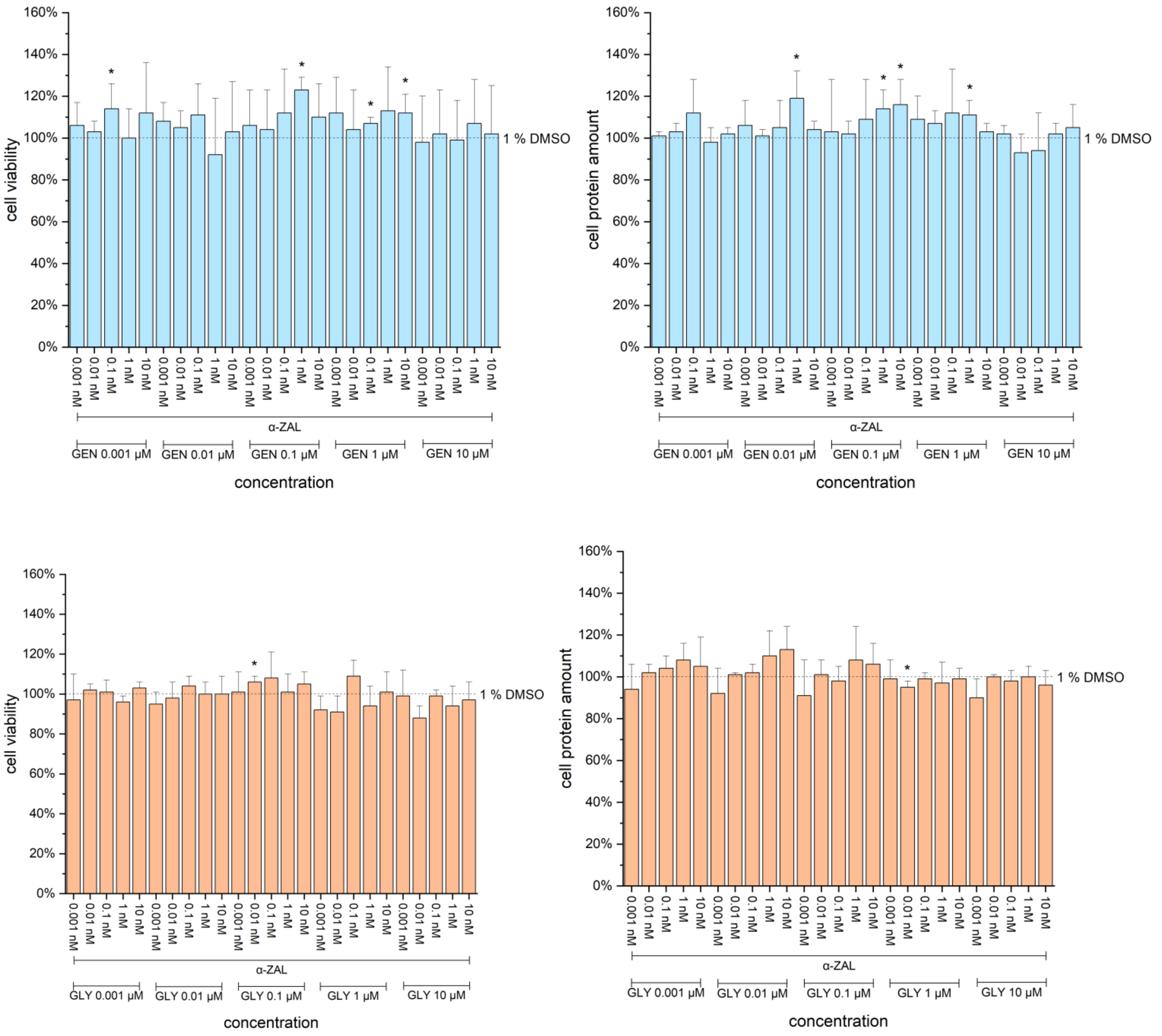

Fig. S3 Effects of the combination of α-zearalanol (α-ZAL) with isoflavone on the cell viability

Impact on the cell viability [%] measured by the CellTiter Blue (CTB) assay (**left**) and on the cell protein amount [%] measured by the sulforhodamine B (SRB) assay (**right**) of different combinations and concentrations after 48 h incubation in Ishikawa cells. Values were referred to the solvent control (1 % DMSO) as 100 %. Results are depicted as mean + standard deviation of at least four biological replicates, calculated from the mean value of three technical replicates. Outliers after Nalimov outlier test were excluded. Significant differences of effects between the solvent control and the incubation solutions were calculated by one-sample Student's *t*-test. Significances are indicated with * ($p < 0.05$). According to its low estrogenicity, GLY was tested in one higher concentration (20 µM) instead of the lowest concentration of 0.001 µM.

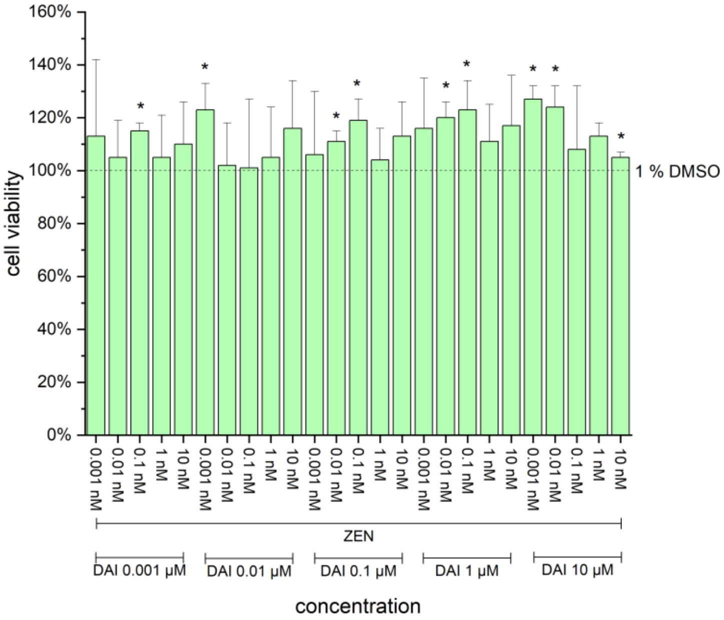
cell viability
160%
140%
120%
100%
80%
60%
40%
20%
0%
1 % DMSO
0.001 nM
0.01 nM
0.1 nM
1 nM
10 nM
ZEN
DAI 0.001 µM
DAI 0.01 µM
DAI 0.1 µM
DAI 1 µM
DAI 10 µM
concentration

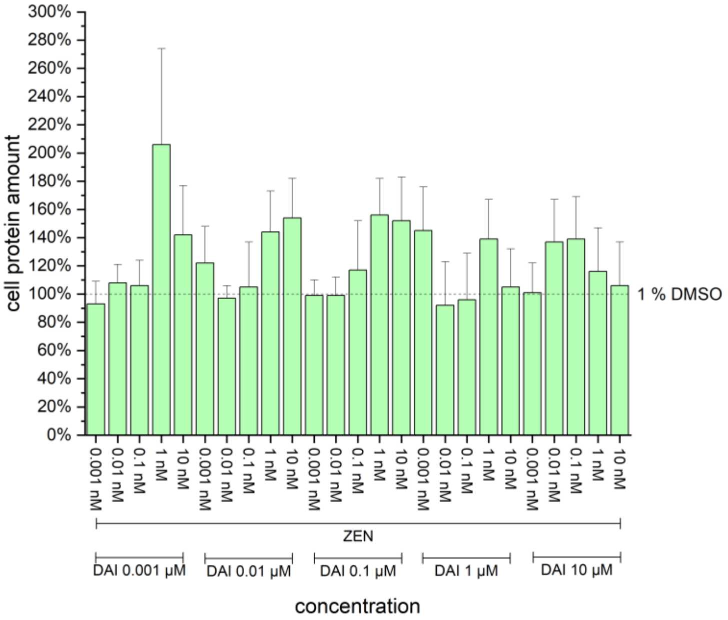
cell protein amount
300%
280%
260%
240%
220%
200%
180%
160%
140%
120%
100%
80%
60%
40%
20%
0%
1 % DMSO
0.001 nM
0.01 nM
0.1 nM
1 nM
10 nM
ZEN
DAI 0.001 µM
DAI 0.01 µM
DAI 0.1 µM
DAI 1 µM
DAI 10 µM
concentration

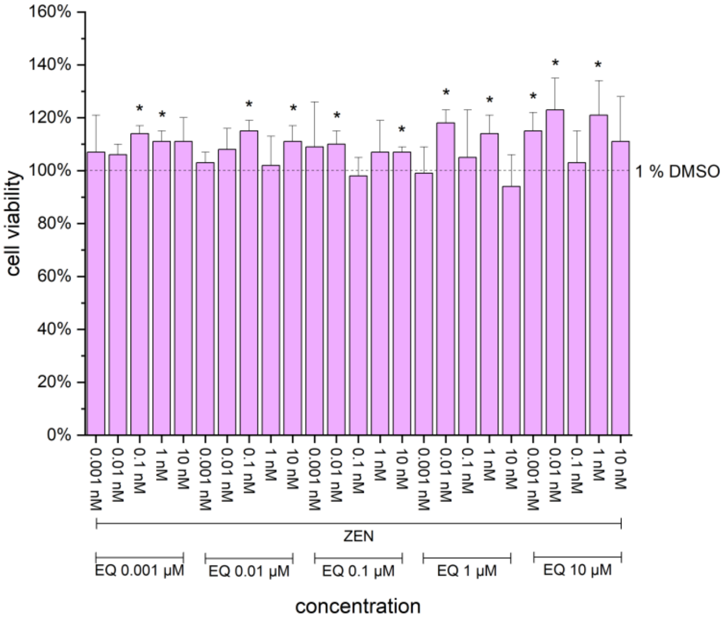
cell viability
160%
140%
120%
100%
80%
60%
40%
20%
0%
1 % DMSO
0.001 nM
0.01 nM
0.1 nM
1 nM
10 nM
ZEN
EQ 0.001 µM
EQ 0.01 µM
EQ 0.1 µM
EQ 1 µM
EQ 10 µM
concentration

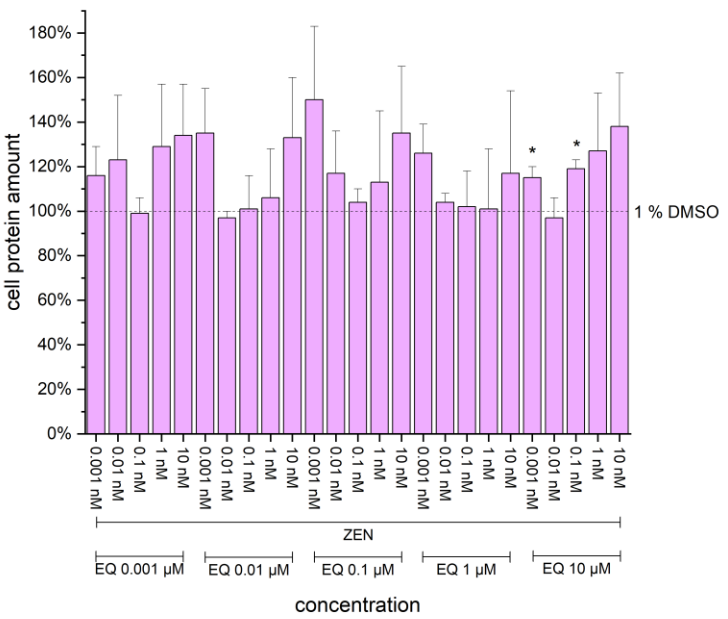
cell protein amount
180%
160%
140%
120%
100%
80%
60%
40%
20%
0%
1 % DMSO
0.001 nM
0.01 nM
0.1 nM
1 nM
10 nM
ZEN
EQ 0.001 µM
EQ 0.01 µM
EQ 0.1 µM
EQ 1 µM
EQ 10 µM
concentration

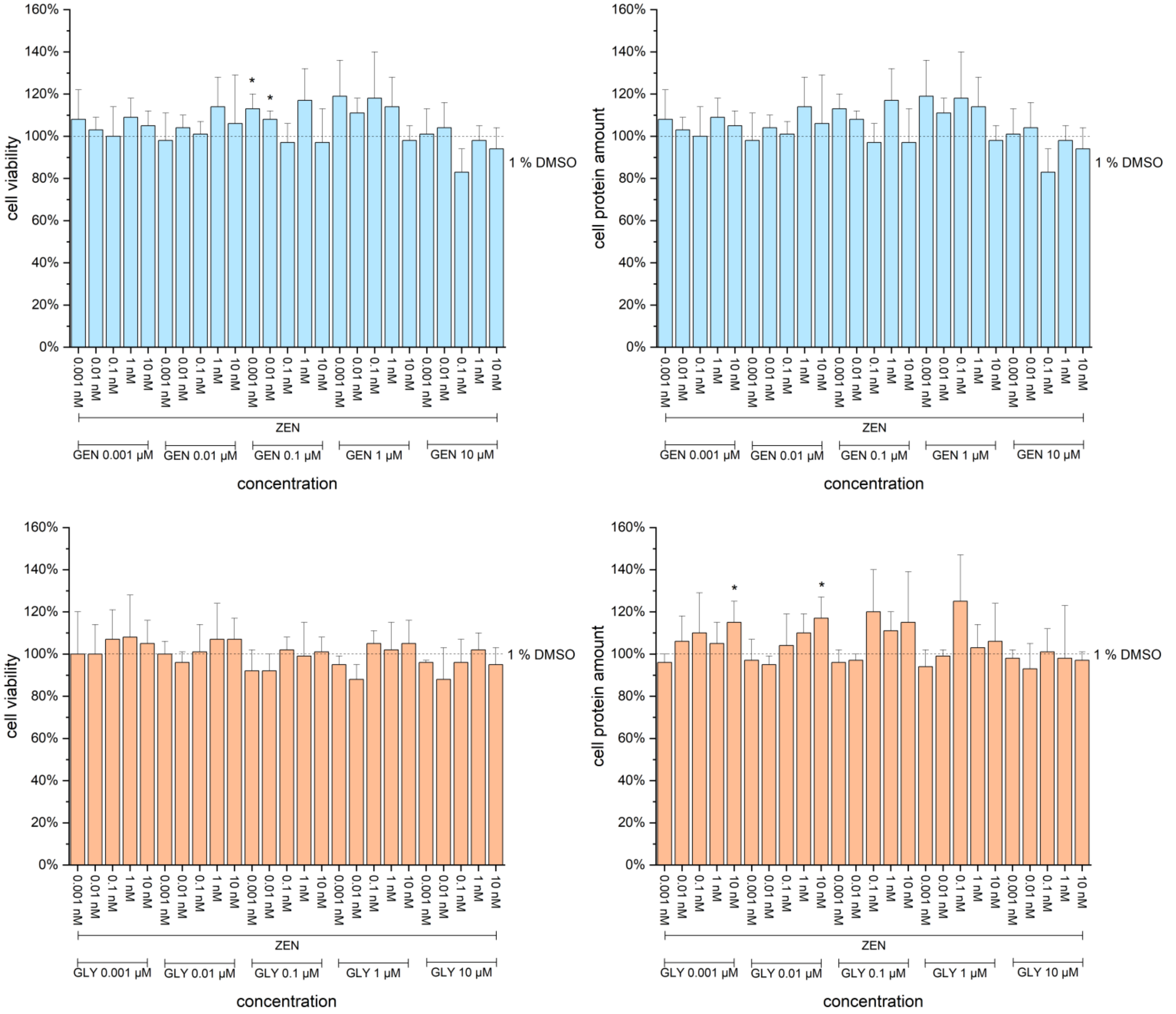

Fig. S4 Effects of the combination of zearalenone (ZEN) with isoflavone on the cell viability

Impact on the cell viability [%] measured by the CellTiter Blue (CTB) assay (**left**) and on the cell protein amount [%] measured by the sulforhodamine B (SRB) assay (**right**) of different combinations and concentrations after 48 h incubation in Ishikawa cells. Values were referred to the solvent control (1 % DMSO) as 100 %. Results are depicted as mean + standard deviation of at least four biological replicates, calculated from the mean value of three technical replicates. Outliers after Nalimov outlier test were excluded. Significant differences of effects between the solvent control and the incubation solutions were calculated by one-sample Student's *t*-test. Significances are indicated with * ($p < 0.05$). According to its low estrogenicity, GLY was tested in one higher concentration (20 µM) instead of the lowest concentration of 0.001 µM.

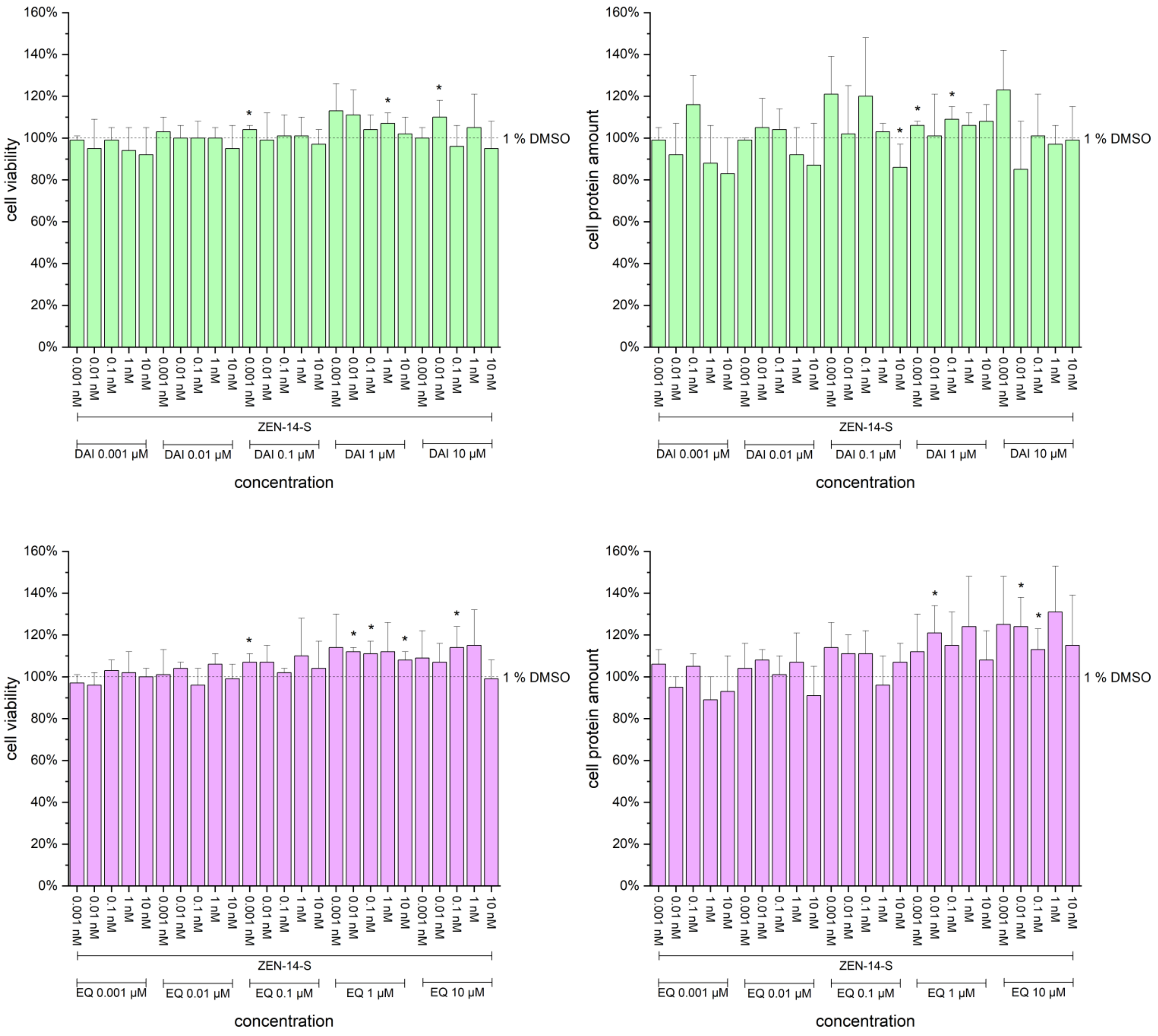

cell viability
cell protein amount
160%
140%
120%
100%
80%
60%
40%
20%
0%
1 % DMSO
0.001 nM
0.01 nM
0.1 nM
1 nM
10 nM
ZEN-14-S
DAI 0.001 µM
DAI 0.01 µM
DAI 0.1 µM
DAI 1 µM
DAI 10 µM
EQ 0.001 µM
EQ 0.01 µM
EQ 0.1 µM
EQ 1 µM
EQ 10 µM
concentration

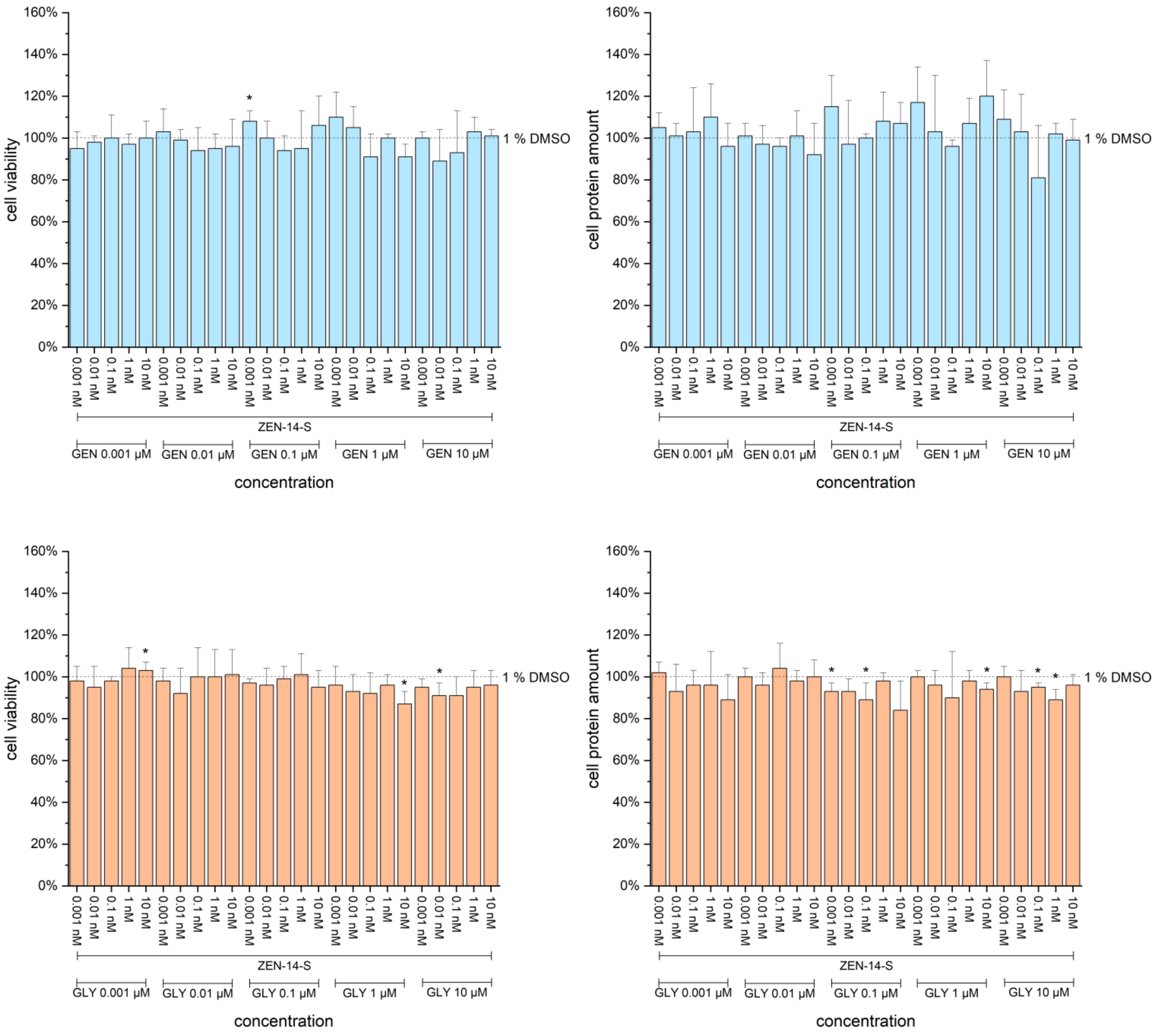

Fig. S5 Effects of the combination of zearalenone-14-sulfate (ZEN-14-S) with isoflavone on the cell viability

Impact on the cell viability [%] measured by the CellTiter Blue (CTB) assay (**left**) and on the cell protein amount [%] measured by the sulforhodamine B (SRB) assay (**right**) of different combinations and concentrations after 48 h incubation in Ishikawa cells. Values were referred to the solvent control (1 % DMSO) as 100 %. Results are depicted as mean + standard deviation of at least four biological replicates, calculated from the mean value of three technical replicates. Outliers after Nalimov outlier test were excluded. Significant differences of effects between the solvent control and the incubation solutions were calculated by one-sample Student's *t*-test. Significances are indicated with * ($p < 0.05$). According to its low estrogenicity, GLY was tested in one higher concentration (20 µM) instead of the lowest concentration of 0.001 µM.
